# TRIM11 facilitates chemoresistance in nasopharyngeal carcinoma by activating the β-catenin/ABCC9 axis via p62-selective autophagic degradation of Daple

**DOI:** 10.1038/s41389-020-0229-9

**Published:** 2020-05-07

**Authors:** Runa Zhang, Si-Wei Li, Lijuan Liu, Jun Yang, Guofu Huang, Yi Sang

**Affiliations:** 1 Jiangxi Key Laboratory of Cancer Metastasis and Precision Treatment, The Third Affiliated Hospital of Nanchang University, Nanchang, People’s Republic of China; 2 Department of Oncology, Tongji Huangzhou Hospital of Huazhong University of Science and Technology, Hubei, People’s Republic of China; 3 Department of Pharmacy, Jiangxi Cancer Hospital, Nanchang, Jiangxi 330029 People’s Republic of China

**Keywords:** Oncogenes, Cell signalling

## Abstract

Chemotherapy resistance is the major cause of nasopharyngeal carcinoma (NPC) treatment failure. Tripartite motif-containing protein (TRIM) family members play important roles in tumor development and chemotherapy failure. Here, based on a screening analysis of 71 TRIM family members by qRT-PCR, we first confirmed that the TRIM11 levels were significantly higher in drug-resistant NPC cells than in non-drug-resistant NPC cells, and high TRIM11 expression predicted poor overall survival (OS) and progression-free survival (PFS). N(6)-Methyladenosine (m6A) was highly enriched in TRIM11 in NPC drug-resistant cells and enhanced its RNA stability. TRIM11 enhanced the multidrug resistance in NPC by inhibiting apoptosis in vitro and promoting cisplatin (DDP) resistance in vivo. TRIM11 associated with Daple and promoted Daple ubiquitin-mediated degradation in a p62-selective autophagic manner, further upregulating β-catenin expression to induce ABCC9 expression by directly binding to the ABCC9 promoter. TRIM11 may regulate NPC drug resistance by positively modulating the Daple/β-catenin/ABCC9 signaling pathway. Thus, TRIM11 may be a potential diagnostic marker and therapeutic target for chemoresistant NPC.

## Introduction

The development of drug resistance is a major cause of cancer mortality^1^. Nasopharyngeal carcinoma (NPC) is particularly prevalent in East and Southeast Asia^2^, and chemotherapy is one of the main treatments for NPC patients based on the National Comprehensive Cancer Network (NCCN) guidelines^3^. Although NPC patients are sensitive to chemotherapy during the initial treatment, they develop acquired resistance shortly after, leading to treatment failure^4^. Studies have shown that altering gene expression promotes chemosensitivity; for example, inhibition of LMP1 could enhance NPC cell sensitivity to chemotherapy^5^. Thus, clarifying the molecular mechanisms of chemotherapy resistance in NPC is important for developing personalized and precise therapeutic approaches.

The tripartite motif-containing protein (TRIM) family, of which many members have E3 ubiquitin ligase activities, is characterized by the presence of three conserved domains, RING, B-Box and coiled-coil (RBCC)^6^. Recent data suggest that members of the TRIM protein family are involved in cancer initiation, progression, and drug resistance^7–11^. A few studies have shown that the TRIM family plays an important regulatory role in NPC. For example, knockdown of TRIM24 induces cell apoptosis and reduces cell viability in NPC^12^, and TRIM29 promotes proliferation and metastasis of NPC^13^. Whether the TRIM family regulates drug resistance in NPC cells remains unclear.

Aberrant Wnt/β-catenin pathway activation, which plays an important role in drug-resistant cancer cells, is frequently observed in NPC^14,15^. Elucidation of the molecular details of the oncogenic activation of Wnt/β-catenin signaling may therefore lead to more effective treatments for patients with NPC. Dvl-associating protein (Daple) with a high frequency of leucine residues was first identified to bind to Dvl^16^. Subsequently, Daple was shown to antagonistically inhibit canonical Wnt responses and enhance the noncanonical Wnt signaling pathway. Mechanistically, in the canonical Wnt signaling pathway, three C-terminal amino acids of Daple bind directly to the region containing the PDZ domain of Dvl and induce Dvl phosphorylation, by which binding to Dvl is responsible for the inhibitory effect of Daple on β-catenin accumulation^16^. In the noncanonical Wnt signaling pathway, Daple serves as a guanine-nucleotide exchange factor (GEF) for the trimeric G protein Gαi and directly binds to FZD receptors^17^. By binding to both FZD and the G protein, Daple enables ligand-activated receptors to recruit and activate Gi and trigger noncanonical Wnt signaling by promoting PI3K-Akt and Rac1 signaling^17,18^. Multiple receptor tyrosine kinases (RTKs) and non-RTKs can phosphorylate Daple, which dissociates Daple: Dvl complexes and augments the ability of Daple to bind and activate Gαi^19^. Daple triggers tumor cell migration but suppresses growth and proliferation; thus, its dysregulation can impact both tumor initiation and progression to metastasis^17^. However, whether Daple regulates cancer drug resistance is unknown.

In this study, we identified high levels of TRIM11 expression in NPC drug-resistant cells and investigated the functional role of TRIM11 in NPC drug resistance. We provide detailed evidence to show that TRIM11-mediated drug resistance depends on activating β-catenin/ABCC9 signaling by facilitating the degradation of Daple.

## Materials and methods

### Cells and reagents

Human NPC cell lines (CNE1 and CNE2) were obtained from the group of Tiebang Kang (Sun Yat‑Sen University Cancer Center, Guangzhou, China). The CNE1 and CNE2 cell lines were cultured in DMEM, and the culture medium was supplemented with 10% fetal bovine serum (Gibco, #10270-106) and maintained at 37 °C in an incubator containing 5% CO_2_. All of the cell lines used in this study were authenticated through short tandem repeat profiling less than 6 months before the start of the experiment when this project was initiated, and the cells were not cultured for more than 2 months. All cell lines were tested for mycoplasma contamination.The cisplatin (DDP)-resistant cell lines CNE2-DDP and CNE1-DDP were generated in our laboratory by continuously exposing the cells to DDP (S1166, Selleck, USA) starting at 0.1 μmol and gradually increasing the concentration to 10 μmol over the course of 6 months until a significant increase in DDP resistance was achieved in the resistant cell lines compared with that in the parental cell lines. CNE2-DDP and CNE1-DDP cells were cultured in the presence of DDP (1.5 μmol) to maintain the DDP-resistant phenotype. 5-Fluoracil (S1209) and docetaxel (S1148) were obtained from Selleck. Actinomycin D (A4448) was obtained from APExBIO. Rapamycin, bafilomycin A1 (Baf-A1), chloroquine (CQ), and ammonium chloride (NH_4_Cl) were purchased from Sigma-Aldrich. MG-132—CAS 133407-82-6 (474790-1MGCN) and cycloheximide-CAS 66-81-9 (#239763-5GMCN) were obtained from Millipore. To induce starvation, cells were washed with PBS and incubated in Earle’s balanced salt solution (EBSS) (Gibco).

### Plasmids

The full-length cDNA of human TRIM11, Daple, METTL3, METTL14, WTAP, FTO, ALKBH5, IGF2BP1, IGF2BP2, IGF2BP3, YTHDF1, YTHDF2, YTHDF3, or YTHDC1 was cloned into the lentivirus vector (pSin-puro), which was used for constructing stable cell lines. The full-length cDNAs of human TRIM11 and its mutants were constructed by inserting an S-Flag-SBP (SFB) tag into the TRIM11 N-terminus in the lentivirus vector plenti-puro (Addgene, #39481). Full-length cDNAs of human Daple and their mutants were constructed by inserting an HA tag into the N-terminus of a pcDNA3.1 (+) vector. Full-length cDNAs of human ubiquitin and its mutants were constructed by inserting an HA tag into the N-terminus of a pcDNA3.1 (+) vector. Full-length cDNAs of human p62, NIX, OPTN, NBR1, Tollip, NDP52, ULK1, ATG13, Beclin-1, ATG14, UVRAG, ATG12, ATG7, ATG5, LC3B, and ATG4B were constructed by inserting an HA tag into the N-terminus of a pcDNA3.1 (+) vector. All recombinant plasmids were verified through DNA sequencing.

### Antibodies

Human anti-GAPDH (#5174), anti-beclin-1 (#3738), anti-LC3B (#3868), anti-flag (#14793), anti-p62 (#5114), and anti-HA (#C29F4) antibodies were obtained from Cell Signaling Technology. Anti-ABCC9 (ab84299) and anti-METTL3 (ab195352) antibodies were obtained from Abcam. An anti-TRIM11 antibody (#HPA028541) was obtained from Sigma-Aldrich, and anti-TRIM11 (for Co-IP detection, PAB24564) was obtained from Abnova Corporation. Anti-LC3 (NB100-2220H) and anti-Beclin1 (NB500-249H) antibodies were obtained from Novus Biologicals. An anti-β-catenin antibody (#66379-1-Ig) was obtained from Proteintech Group, Inc. An anti-Daple antibody (#A302-951A) was obtained from Bethyl Laboratories, Inc. An anti-IGF2BP2 antibody (sc-377014) was obtained from Santa Cruz Biotechnology.

### Stable lines

These procedures were performed as previously described^20^. Briefly, a recombinant lentivirus plasmid verified by DNA sequencing was cotransfected with the pMD2.G and psPAX2 plasmids into 293T cells with Lipofectamine 2000 for 48 h. The recombinant viruses were subsequently collected and added to cells cultured with 8 μg/ml polybrene for 24 h. The stable lines were selected with 1 μg/ml puromycin for 2 weeks.

### Generation of knockout or knockdown cell lines

To generate TRIM11-KO, BECN1-KO, LC3B-KO, or Daple knockdown cells, target sequences were cloned into pLentiCRISPRv2 by cleavage with BsmBI as previously described^21^. Then, the target plasmids were cotransfected with the pMD2.G and psPAX2 plasmids into HEK-293T cells for 48 h. The recombinant viruses were subsequently collected and added to cells cultured with 8 μg/ml polybrene for 24 h. The stable lines were selected with 1 μg/ml puromycin for two weeks. The clones were then picked in 96-well plates and genotyped. KOs were validated by Sanger sequencing and western blots when the corresponding antibody was available. The following sgRNA sequences were used: BECN-1: 5′-GGGTCTCTCCTGGTTTCGCC-3′; LC3B: 5′-TTCAAGCAGCGCCGCACCTT-3′; TRIM11: 5′-TGCGTTGCTGTTCCAAG CCC-3′. Daple: 5′-GCAGCATGGACGTGACAGTCT-3′.

### Flow cytometry

Apoptosis analysis was conducted with an Annexin V-FITC Apoptosis Detection Kit (KeyGen Biotech, China) according to the manufacturer’s protocol. The percentage of apoptotic cells was determined using a FACS flow cytometer equipped with software (BD).

### In vivo ubiquitination assay

This procedure was performed as previously described^22^. Briefly, CNE2 cells were transfected with the indicated plasmids for 24 h and were treated with 1 μM Baf-A1 for 6 h prior to harvesting. The cells were lysed in RIPA buffer with a protease inhibitor cocktail (Sigma-Aldrich) and a phosphatase inhibitor cocktail (Calbiochem). Flag-Daple was immunoprecipitated using anti-Flag agarose for 12 h at 4 °C. Polyubiquitinated Daple was detected using an anti-HA antibody.

### m6A RNA methylation quantification

Total m6A levels of the extracted RNA were detected by an m6A RNA Methylation Quantification Kit (P-9005, Epigentek Group, Inc., USA) according to the manufacturer’s instructions. Poly-A purified RNA (200 ng) was used for each sample analysis.

### RNA extraction and reverse transcription-quantitative polymerase chain reaction (qRT-PCR)

These procedures were performed as previously described^20,23^. Briefly, total RNA was isolated using TRIzol^®^ Reagent (Invitrogen; Thermo Fisher Scientific, Inc.) according to the manufacturer’s instructions. Reverse transcription for first-strand cDNA was synthesized using the RevertAid^TM^ First Strand cDNA Synthesis Kit (cat. no. 6210A, TaKaRa Bio, Inc., Otsu, Japan). Subsequently, qPCR was performed in a CFX96 Real-Time PCR Detection system (Bio-Rad Laboratories, Inc., Hercules, CA, USA) using SYBR^®^ Green mix (Tiangen Biotech Co., Ltd., Beijing, China). The qPCR thermal cycling conditions were as follows: a denaturation step at 95 °C for 15 min and 40 cycles of denaturation at 95 °C for 15 s, annealing at 60 °C for 30 s and elongation at 72 °C for 30 s. The amplified products were examined using the 2^−∆∆^Cq method^24^, and each sample was calibrated to the expression levels of the housekeeping gene GAPDH. The primers are shown in Supplementary Table 1.

### RNA immunoprecipitation

The experiments were carried out using the Magna RIP RNA-Binding Protein Immunoprecipitation Kit (Millipore, Bedford, MA) according to the manufacturer’s instructions. qRT-PCR was performed to analyze TRIM11 RNA in the precipitates after the antibody was recovered.

### Cell viability assays

Cells were seeded in 96-well plates at a density of 5000 cells/well and treated with DDP or 5-fluorouracil (5-FU) for 24 h. Ten microliters of CCK-8 reagent was then added to each well, followed by incubation for 1.5 h. The absorbance value (optical density) of each well was measured at 450 nm using a microplate reader (ENSPIRE, PE).

### Colony formation assays

Cells in this study were plated in six-well culture plates at a density of 5 × 10^3^ cells/well and treated with DDP for 24 h. Each group consisted of three wells. The cells were incubated for 15 days at 37 °C, washed twice with PBS, fixed with 10% formalin for 15 min, and stained with 0.1% crystal violet for 60 min. A cluster containing ≥50 cells was counted as a colony, and cell counting was performed with a light microscope.

### Western blotting and immunoprecipitation

Western blotting and immunoprecipitation were performed as described previously^20^. Briefly, cells were lysed in RIPA buffer (150 mM NaCl, 0.5% EDTA, 50 mM Tris, 0.5% NP40) at 4 °C for 30 min, and clarified lysates were resolved by sodium dodecyl sulfate (SDS)-polyacrylamide gel electrophoresis and transferred to polyvinylidene difluoride membranes for western blotting using ECL detection reagents (Beyotime, Co., Haimen, Jiangsu, China). Alternatively, clarified supernatants were first incubated with anti-HA-agarose (Sigma-Aldrich), anti-FLAG-agarose (Sigma-Aldrich) or S protein beads (Novagen) for 2 h to overnight at 4 °C, and precipitates were washed four times with mammalian cell lysis buffer (MCLB). To investigate the interaction between TRIM11 and Daple at the endogenous level, the clarified supernatants were incubated with anti-TRIM11 or anti-Daple antibodies for 2 h at 4 °C. Then, protein A/G agarose was added for overnight, and precipitates were washed four times with MCLB and analyzed by western blotting. For the cycloheximide (CHX) chase assay, cells were treated with 20 μg/ml CHX for 0, 2, 4, and 6 h and then lysed and analyzed by western blotting.

### Tandem affinity purification of the TRIM11-associated protein complex

For affinity purification, CNE2 cells stably expressing SFB-TRIM11 proteins were lysed with NETN buffer for 20 min. Crude lysates were removed by centrifugation at 14,000 rpm at 4 °C for 10 min and then incubated with streptavidin-conjugated beads (Amersham) for 2 h at 4 °C. The immunocomplexes were washed three times with NETN buffer, and then bead-bound proteins were eluted with NETN buffer containing 2 mg/ml biotin (Sigma-Aldrich). The eluates were incubated with S protein beads (Novagen). The immunocomplexes were again washed three times with NETN buffer and subjected to SDS-PAGE. Protein bands were excised and digested, and the peptides were analyzed by mass spectrometry.

### RNA interference

Approximately, 6 × 10^5^ cells/well were seeded in 6 cm culture plates the day prior to transfection. Transfection was performed according to the manufacturer’s instructions, and the cells were transfected with 50 nM siRNA using Lipofectamine RNAiMAX Transfection Reagent (Invitrogen; Thermo Fisher Scientific, Inc.). The effective siRNA oligonucleotides targeting ABCC9, IGF2BP2 and p62 were as follows: ABCC9: 5′-TTGCGCCAATTCAGTACTTTA-3′; IGF2BP2: 5′-AGTGAAGCTGGAAGCGCATAT-3′; p62: 5′-GCATTGAAGTTGATATCGAT-3′.

### RNA sequencing

RNA was extracted from CNE2-DDP or CNE2-DDP-TRIM11 KO cells using the RNeasy Mini Kit (Qiagen) according to the manufacturer’s protocol. RNA samples were quantified using a Qubit 2.0 Fluorometer (Life Technologies, Carlsbad, CA, USA), and RNA integrity was assessed with a 4200 TapeStation (Agilent Technologies, CA, USA). The RNA sequencing library was prepared using an NEBNext Ultra RNA Library Prep Kit for Illumina following the manufacturer’s instructions (NEB, Ipswich, MA, USA). Sequencing was performed on the Illumina HiSeq platform using a 2 × 150 paired-end (PE) configuration with 20 million reads per sample. All datasets were deposited in GEO (https://www.ncbi.nlm.nih.gov/geo/), and the accession number is GSE140421.

### Luciferase assay

The assay was carried out as described previously. Briefly, 293T cells were seeded in 12-well plates at a density of 3 × 10^5^ cells/well and transfected with 0.8 μg of a promoter luciferase plasmid. To normalize the transfection efficiency, the cells were cotransfected with 8 ng Renilla luciferase. After transfection for 48 h, the luciferase activity was measured using the Dual-Luciferase Assay Kit (Promega, Madison, WI, USA). Three independent experiments were performed.

### Immunofluorescence staining

Cells grown on coverslips were fixed using 4% paraformaldehyde solution for 15 min at room temperature, washed with 0.1% NP40/PBS and then extracted with buffer containing 0.5% Triton X-100 for 5 min. Samples were blocked with 5% goat serum and incubated with primary antibody overnight at 4 °C. Antibodies against Flag (1:100), HA (1:100), or β-catenin (1:200) were used to detect the Flag-TRIM11, HA-Daple, and endogenous β-catenin proteins, respectively. Samples were washed three times with 0.1% NP40/PBS and incubated with the secondary antibody at room temperature for 1 h. Cells were then counterstained with Hoechst 33342 at room temperature for 5 min to visualize nuclear DNA. The coverslips were mounted onto glass slides and visualized by a fluorescence microscope.

### Chromatin immunoprecipitation (ChIP) assay

The ChIP assay was performed using the ChIP kit (cat. no. 53008, Active Motif, Carlsbad, CA, USA) as described previously^20^. Briefly, to fix the cells, Complete Cell Fixative Solution (included in the kit) was added to the existing culture medium of cells at 80% confluence at room temperature, and the fixation reaction was stopped by adding Stop Solution (included in the kit) to the existing culture medium. The cells were collected by centrifugation at 1000×*g* for 5 min at 4 °C. Subsequently, the nuclear pellet was resuspended in ChIP buffer (included in the kit). The cell lysate was subjected to shearing using a sonication instrument (Ningbo Scientz Biotechnology Co., Ltd., Ningbo, China) to a fragment length of 200–500 bp. Total genomic DNA (input) was quantified, and 20 μg of chromatin from each sample was immunoprecipitated overnight at 4 °C with 5 μg anti‑β-catenin (ab32572, Abcam) or normal IgG as a negative control. Then, nucleosome complexes were isolated with protein G agarose beads for 3 h at 4 °C. Bound DNA‑protein complexes were eluted, and cross‑links were reversed after a series of washes using the washing reagent contained in the ChIP kit. Purified DNA was resuspended in TE buffer. Subsequently, PCR was performed using PrimeSTAR^®^ Max DNA Polymerase (cat. no. R045A; TaKaRa Bio, Inc.). The qPCR thermal cycling conditions included a denaturation step at 94 °C for 2 min and 35 cycles of denaturation at 98 °C for 10 s, annealing at 60 °C for 15 s and elongation at 72 °C for 30 s. The primers for ABCC9‑ChIP were as follows: forward, 5′‑GTTATAGCCATGGTAGCTAGCTAAC‑3′; reverse, 5′‑TTAGGGCTTTA TCATCATCTAGAGC‑3′. The primers for ABCC9‑control-ChIP were as follows: forward, 5′‑TTTGCTCATCTCCCATCTGTTTG‑3′; reverse, 5′‑CAGGATTG CGGCTTCTACTCTTA‑3′.

### Animal experiments

All animal studies were performed in accordance with protocols approved by Jiangxi University of Traditional Chinese Medicine (Nanchang, China). The mice were maintained in specific pathogen-free conditions at a temperature of 20–25 °C and 50–70% humidity under a light/dark cycle of 12 h with free access to water and food. A total of 28 male athymic nude mice at 4 weeks of age were obtained from Shanghai Institutes for Biological Sciences, Chinese Academy of Sciences (Shanghai, China). A total of 2 × 10^6^ cells was mixed with 0.2 ml PBS (pH 7.4) and 30% (v/v) Matrigel matrix (BD Biosciences). Suspensions were injected subcutaneously into the flanks of the randomly assigned nude mice, which were monitored over 4 weeks. An intraperitoneal injection of DDP (3 mg/kg per week for 2 weeks) was administered every 3 days; the control group received 200 μl of 0.1% DMSO.

### Study approval

The use of human NPC tissues was reviewed and approved by the Ethical Committee of The Third Affiliated Hospital of Nanchang University (Nanchang, China). Written informed consent was obtained from all patients. A total of 20 tumor specimens were collected from patients with NPC (median age, 46 years; age range, 35–88 years; male:female ratio, 2:3), and resection occurred between January 2015 and December 2017.

### Statistical analysis

Data from independent experiments are presented as the means ± SDs. Statistical analysis between two groups was performed by Student’s *t* test (two-tailed), and statistical analysis among multiple groups was conducted by one-way ANOVA with SPSS version 18.0 (IBM Analytics, USA). All experiments in vitro were performed independently at least three times and in triplicate each time, and the mean values of three experiments are shown. A *P* value < 0.05 was considered statistically significant in all cases (*), and a *P* value < 0.01 was considered strongly statistically significant in all cases (**).

## Results

### Result 1. TRIM11 expression is associated with a malignant NPC subtype

**Fig. 1:**
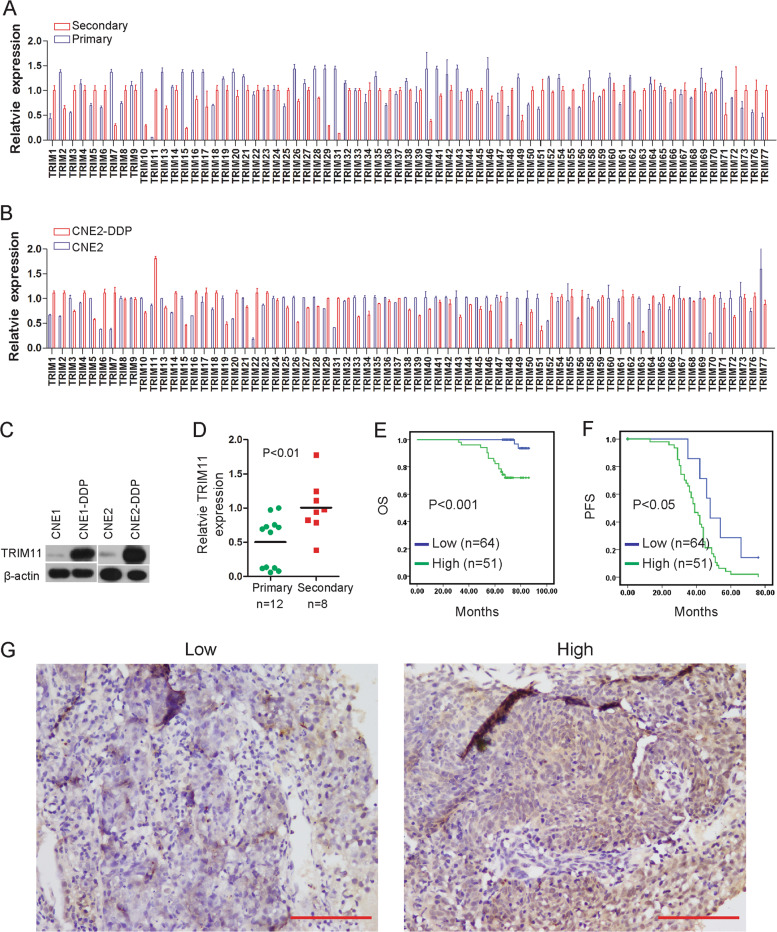
TRIM11 is expressed at increased levels in NPC drug-resistant cells and predicts a poor outcome. **a**, **b** The mRNA levels of TRIM family members were determined via qRT-PCR in NPC primary tissues vs NPC secondary tissues (**a**) and CNE2 vs. CNE2-DDP cells (**b**). GAPDH was used as an internal control. Data are presented as the mean ± standard error (*n* = 3). **c** The protein level of TRIM11 determined via western blot analysis in CNE1 vs. CNE1-DDP and CNE2 vs. CNE2-DDP cells. β-actin was used as an internal control. **d** The mRNA level of TRIM11 was determined via qRT-PCR in NPC primary tissues vs NPC secondary tissues. **e**, **f** OS (**e**) and PFS (**f**) curves were generated based on the TRIM11 protein expression status of 115 paraffin-embedded NPC tumor tissues. *P* < 0.05. **g** IHC of clinical NPC samples showed the expression of TRIM11: low level (left) and high level (right). Scale bar, 50 µm.

Concurrent/adjuvant DDP-based chemoradiotherapy is regarded as the standard of treatment for NPC patients. To explore the roles of TRIMs in drug-resistant NPC cells, we used qRT-PCR to screen the pair of NPC primary tumor specimens (primary) and recurrent NPC specimens (secondary) in the same patient and two cell lines of CNE2 and CNE2-DDP with low and high drug resistance, respectively, as shown in Fig. 1a. Although many of these gene expression levels were different between the primary tumor and the secondary tumor and/or those of CNE2 and CNE2-DDP, only TRIM11 was simultaneously upregulated, and the fold change was >twofold higher in CNE2-DDP and secondary NPC tissues than in CNE2 and primary NPC tissues. Then, western blotting was performed and showed that the protein level of TRIM11 increased in CNE1-DDP and CNE2-DDP cells compared with that in CNE1 and CNE2 cells, respectively (Fig. 1c). To further examine the expression of TRIM11 in patient tissues, we collected 12 NPC primary tissues and 8 NPC secondary tissues, and the real-time PCR results showed that TRIM11 was significantly increased in secondary tissues (Fig. 1d).

To evaluate the prognostic value of TRIM11 expression, we performed immunohistochemistry (IHC) staining for TRIM11 in a set of tissue microarrays containing 115 NPC samples (Supplementary Table S2). Kaplan–Meier analysis showed that increased TRIM11 expression was significantly correlated with decreased overall survival (OS) and progression-free survival (PFS) in patients with NPC (Fig. 1e–g). Moreover, to validate the results of our survival analysis, we applied multivariate Cox regression models to verify our results, suggesting that TRIM11 was a unique indicator of poor survival prognosis (Supplementary Table S3). These results confirmed that upregulation of TRIM11 is clinically relevant and may be used as an independent prognostic predictor for NPC patients.

### Result 2. The m6A modification is enriched in TRIM11 in NPC drug-resistant cells and improves TRIM11 transcript stability

**Fig. 2:**
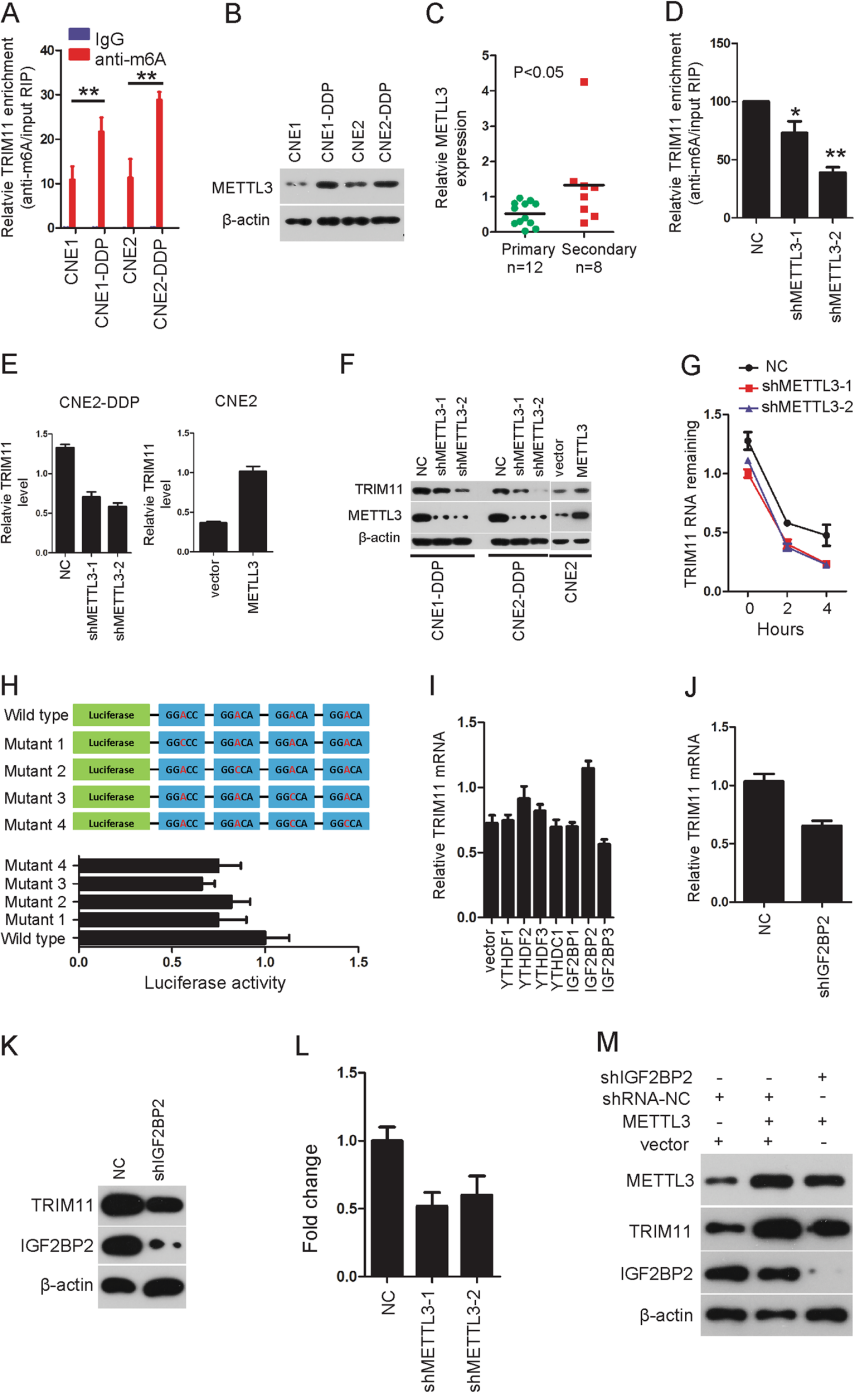
METTL3-mediated m6A modification caused the upregulation of TRIM11 via IGF2BP2 in NPC drug-resistant cells. **a** The m6A methylation level of TRIM11 in CNE1, CNE1-DDP, CNE2, and CNE2-DDP cells was determined by MeRIP-qPCR assays, **P < 0.01. **b** The protein level of METTL3 was detected in the indicated cells. β-actin was used as an internal control. **c** The mRNA level of METTL3 was determined via qRT-PCR in NPC primary tissues vs. NPC secondary tissues. GAPDH was used as an internal control. **d** Changes in m6A-modified TRIM11 mRNA after knocking down METTL3 in CNE2-DDP cells. *P < 0.05, **P < 0.01. **e**, **f** The mRNA and protein levels of TRIM11 were detected after overexpressing or knocking down METTL3. **g** The RNA stability of TRIM11 was detected in the indicated cells. Cells were treated with 5 mg/ml actinomycin D, and RNA was isolated at 0, 2, and 4 h. *P < 0.05. **h** WT or mutant m6A consensus sequences within TRIM11 mRNA were fused with a firefly luciferase reporter. CNE2 cells stably transfected with an empty vector or TRIM11 were transfected with a luciferase reporter plasmid as described above. Luciferase activity was measured as described in the “Materials and methods” section. **i** The mRNA of TRIM11 was detected after overexpressing the m6A direct readers, and only IGF2BP2 increased the mRNA level of TRIM11. **j**, **k** Silencing IGF2BP2 by siRNA resulted in decreased mRNA and protein levels of TRIM11 in CNE2-DDP cells. **l** RIP-qPCR using an anti-IGF2BP2 antibody showed the affinity of TRIM11 mRNA to IGF2BP2 in differently modified METTL3-expressing cells. **m** TRIM11 and IGF2BP2 mRNA levels were determined by qRT-PCR in CNE2 cells transfected with METTL3 and/or si-IGF2BP2.

Since m6A modifications in mRNA could promote transcript stability^25^, we first used the online bioinformatics tool m6Avar (http://m6avar.renlab.org/) to analyze the methylation status of TRIM11 m6A, and multiple m6A modification sites (RRACH) in TRIM11 mRNA were found. Next, methylated RNA immunoprecipitation (Me-RIP) assays were performed and showed that the m6A level of TRIM11 was higher in CNE1-DDP and CNE2-DDP cells than in CNE1 and CNE2 cells (Fig. 2a). As METTL3 is a core component of the m6A methyltransferase complex, western blotting was performed and showed that METTL3 was overexpressed in CNE1-DDP and CNE2-DDP cells compared with that in CNE1 and CNE2 cells (Fig. 2b), respectively. In addition, using qRT-PCR, we found that METTL3 was significantly increased in secondary tissues compared with that in primary tissues (Fig. 2c), indicating that METTL3 may participate in drug resistance. Indeed, overexpression of METTL3 could promote cell viability under a series of doses DDP (Supplementary Fig. 1). To determine whether METTL3 targeted TRIM11 mRNA for m6A modification, METTL3 knockdown was performed and caused a decrease in the m6A level of TRIM11 RNA (Fig. 2d). Knockdown or overexpression of METTL3 significantly decreased or increased the RNA and protein levels of TRIM11 (Fig. 1e, f), respectively. However, the mRNA level of TRIM11 showed no significant changes after stably overexpressing the methyltransferase complex components METTL14 and WTAP or the demethylases FTO and ALKBH5 (Supplementary Fig. 2). Furthermore, the mRNA half-life of TRIM11 was shorter after silencing METTL3 when the cells were treated with actinomycin D (Fig. 2g). Then, we constructed luciferase reporters containing either wild-type (WT) or mutant TRIM11 to address which site is essential for the effect of METTL3-mediated m6A modification on TRIM11 expression. m6A modification was abrogated on mutant TRIM11 because of the replacement of the adenosine base by a cytosine in m6A consensus sequences (RRACH) (Fig. 2h). A luciferase reporter assay showed that the transcriptional level of WT TRIM11, but not that of the mutant, significantly increased after overexpressing METTL3 (Fig. 2h). The above results suggest that METTL3-mediated TRIM11 m6A modification can regulate the stability of TRIM11 RNA.

YTHDF1, YTHDF2, YTHDF3, YTHDC1, IGF2BP1, IGF2BP2, and IGF2BP3 have been identified as direct m6A readers that affect the translation, stability, and/or splicing of target mRNAs^26,27^. Overexpression of YTHDF1, YTHDF2, YTHDF3, YTHDC1, IGF2BP1, IGF2BP2, or IGF2BP3 showed that only IGF2BP2 increased the mRNA and protein levels of TRIM11 (Fig. 2i; Supplementary Fig. 3). Reciprocally, silencing IGF2BP2 reduced the expression of TRIM11(Fig. 2j, k). Indeed, RIP-qPCR using an anti-IGF2BP2 antibody showed a dramatically reduced affinity of IGF2BP2 for TRIM11 mRNA in METTL3-silenced CNE1-DDP cells (Fig. 2l). Consistently, we found that siRNA-induced IGF2BP2 knockdown could partially counteract the effects of elevated METTL3 on the expression of TRIM11 (Fig. 2m). In summary, METTL3-mediated m6A modifications of TRIM11 promoted its transcript stability via the m6A-IGF2BP2-dependent pathway, which may at least partially increase the expression of TRIM11 in drug-resistant NPC cells.

### Result 3. TRIM11 strengthens chemoresistance in NPC

**Fig. 3:**
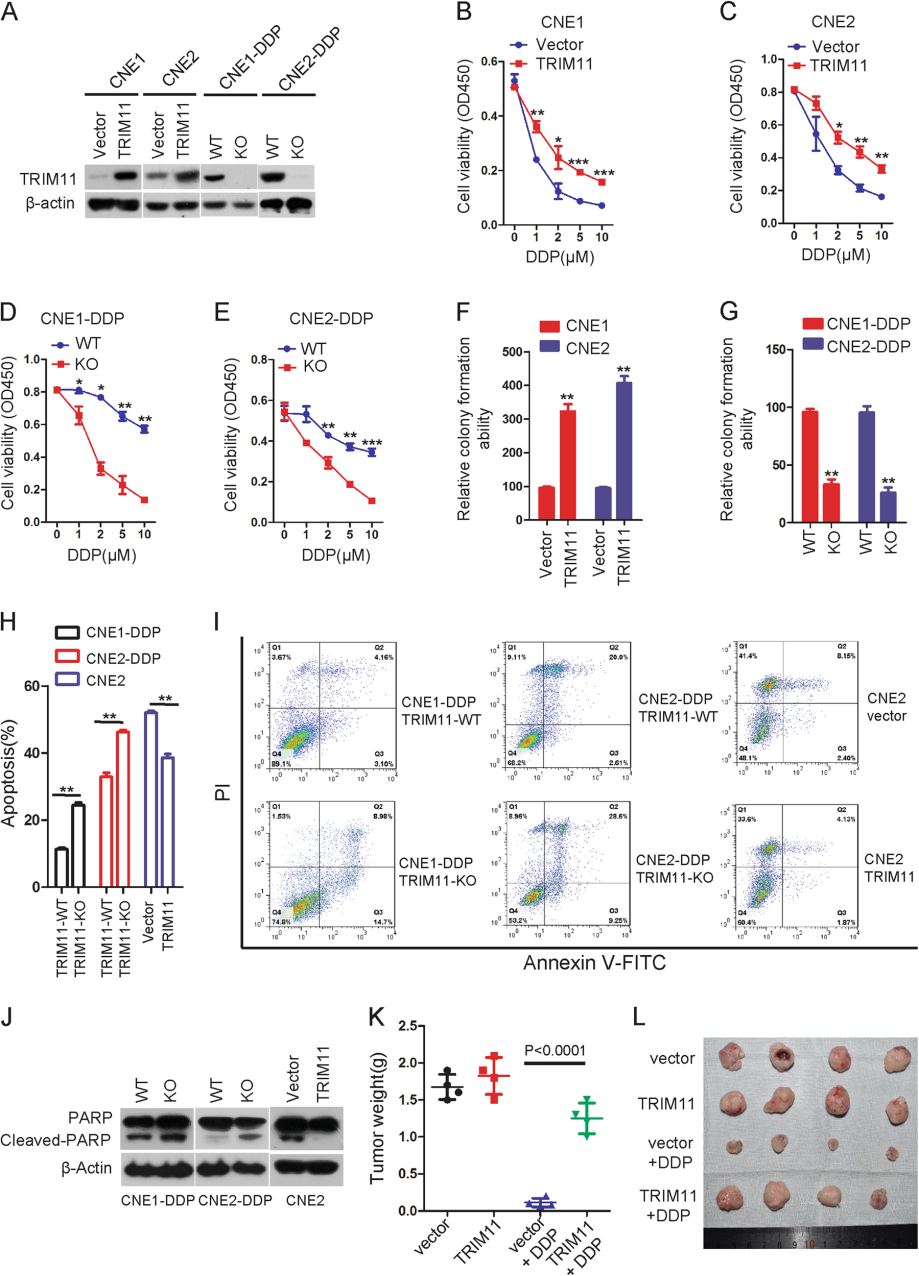
TRIM11 strengthens chemoresistance in NPC by suppressing DDP-induced apoptosis. **a** Generation of stable cell lines in CNE1 and CNE2 cells in which TRIM11 was overexpressed was confirmed through western blotting, and KO of TRIM11 in CNE1-DDP and CNE2-DDP cells was confirmed through western blotting. β-actin was used as the internal control. **b**–**e** The indicated cells were treated with the indicated concentrations of DDP for 24 h, and cell viability was assessed with the CCK-8 assay. The bars correspond to the mean ± standard error (*n* = 3), and the *P* value was calculated using Student’s *t* test. *P < 0.05; **P < 0.01, ***P < 0.001. **f**, **g** The colony formation of the indicated stable cell lines in vitro was measured, as described in the “Materials and methods” section. **P < 0.01. **h**, **i** The indicated stable cell lines were treated with DDP for 24 h and then subjected to annexin V-FITC and propidium iodide (PI) staining. Cell apoptosis was evaluated through FACS. The bars correspond to the mean ± standard error (*n* = 3), and the *P* value was calculated using Student’s *t* test. **P < 0.01. **j** The indicated stable cells were treated with 10 μM DDP for 24 h and subjected to western blotting. **k**, **l** A xenograft model consisting of nude mice with CNE2-DDP and CNE2-DDP-TRIM11-KO cells was injected into the armpits of 4-week-old mice. Images of tumors from the mice (**k**). Mean tumor weights were calculated (**l**). The results are presented as the means ± SDs. ***P < 0.001.

The above results led us to wonder whether increased TRIM11 levels play a key role in DDP-resistant NPC cells. First, we constructed stable cell lines with either ectopic expression of TRIM11 in CNE1 and CNE2 cells or KO of TRIM11 by CRISPR/Cas9 gene editing in CNE1-DDP and CNE2-DDP cells (Fig. 3a). We then investigated the association of TRIM11 expression with DDP sensitivity in vitro. The stable cell lines were treated with different concentrations of DDP, and the cell survival assays indicated that the ectopic expression of TRIM11 significantly improved cell viability and colony formation abilities under DDP exposure; in contrast, deletion of TRIM11 inhibited the cell viability and colony formation abilities (Fig. 3b–g). An important feature of drug resistance is an increased capacity to prevent apoptosis when treated with drugs; thus, the annexin V-propidium iodide assay was performed to evaluate the effect of TRIM11 on cell apoptosis. As shown in Fig. 3h, i, compared with the control cells, TRIM11-KO NPC cells had an increased apoptosis rate. In contrast, TRIM11 overexpression reduced NPC cell apoptosis. Moreover, KO or overexpression of TRIM11 increased or decreased the cleavage of PARP, a well-known marker of cell apoptosis, respectively (Fig. 3j). 5-FU is also one of the most effective chemotherapeutic drugs used for the treatment of NPC. To investigate whether TRIM11 promotes multidrug resistance in NPC, CNE1, and CNE2 cells expressing a vector or TRIM11 were treated with 5-FU and the results demonstrated that overexpression of TRIM11 promoted chemoresistance to 5-FU in NPC in vitro (Supplementary Fig. 4a, c).

To confirm whether TRIM11 enhances NPC cell drug resistance in vivo and to demonstrate the promotive effects of TRIM11, an in vivo tumorigenesis study was performed by inoculating CNE2-vector cells or CNE2-TRIM11 cells into nude mice. Mice treated with DDP or saline were sacrificed 2 weeks after inoculation, and the results showed that the TRIM11 ectopic group (average tumor weight 1.25 g) exhibited significantly enhanced drug resistance compared with the vector group (average tumor weight 0.1125 g) (*P* < 0.001; Fig. 3k, l). These results suggest that TRIM11 promotes NPC drug resistance.

### Result 4. TRIM11 upregulates ABCC9 expression to facilitate chemoresistance

**Fig. 4:**
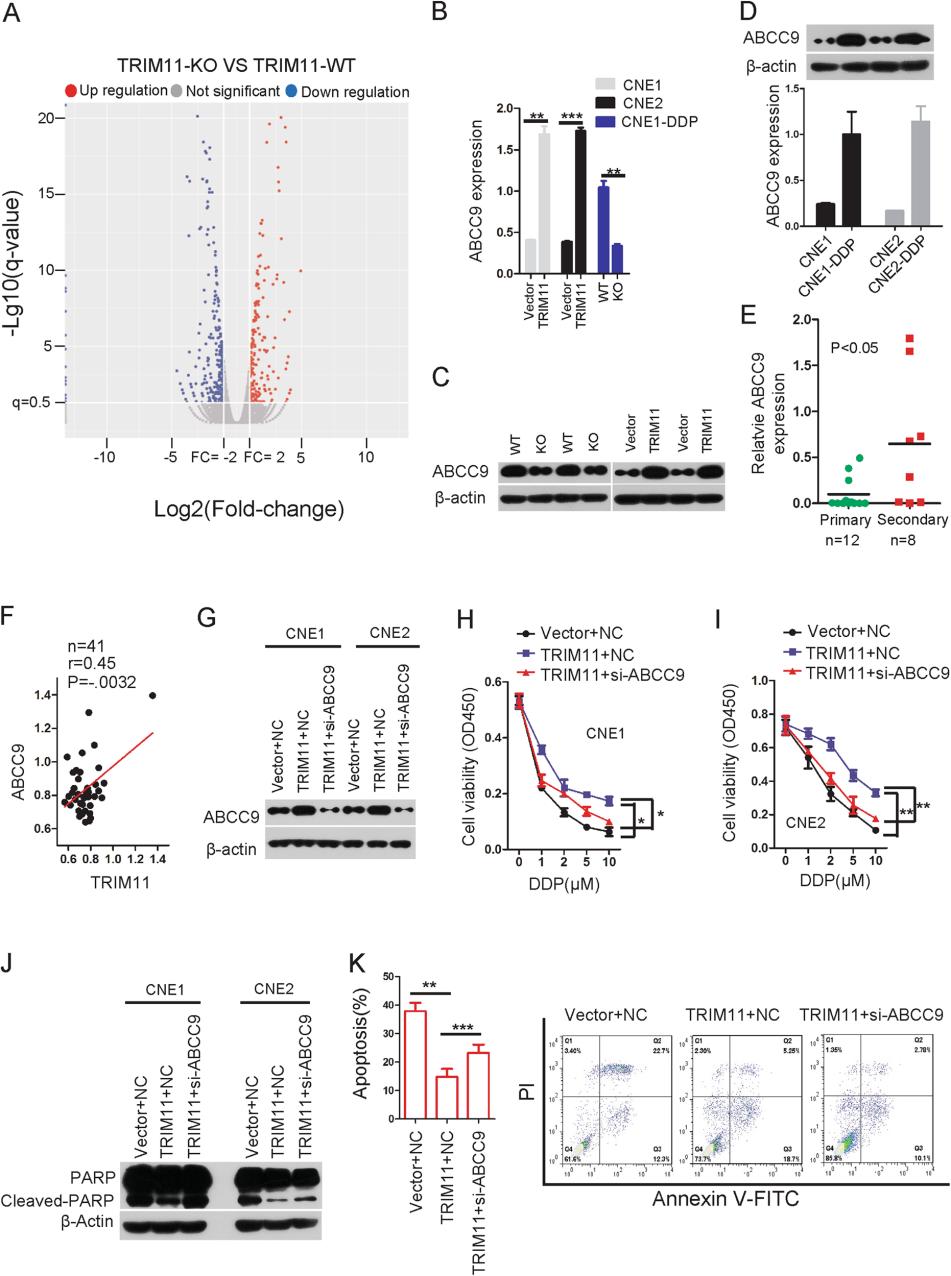
TRIM11 promoted the expression of ABCC9 in NPC cells. **a** The expression profiles for the human transcriptome in the indicated cell lines using RNA sequencing. **b**, **c** The mRNA levels of ABCC9 were determined in the indicated cell lines by qRT-PCR. GAPDH was used as a loading control for qRT-PCR. Data are described as the mean ± SD, Student’s *t* test, *P < 0.05, ***P < 0.01. **d**, **e** The mRNA and protein levels of ABCC9 in the indicated cell lines were determined by qRT-PCR, and GAPDH was used as a control for qRT-PCR. Data are described as the mean ± SD. **f** A significant positive correlation between TRIM11 expression and ABCC9 expression was observed in the tissues. The raw data were obtained from the Oncomine database. **g** The protein level of ABCC9 was detected after knocking down ABCC9 by siRNA or negative control (NC) in NPC cells. **h**, **i** The cell viability of the indicated cell lines in vitro was determined by CCK8 assays. Data are described as the mean ± SD, Student’s *t* test, *P < 0.05, **P < 0.01. **j** The indicated cells were treated with 10 μM DDP for 24 h and subjected to western blotting. **k** The indicated cells were treated with DDP for 24 h and then subjected to annexin V-FITC and propidium iodide (PI) staining. Cell apoptosis was evaluated through FACS. The bars correspond to the mean ± standard error (*n* = 3), and the *P* value was calculated using Student’s *t* test. **P < 0.01, ***P < 0.001.

To elucidate the mechanism underlying the chemoresistance-promoting effect of TRIM11 in NPC, RNA sequencing was performed on TRIM11-KO cells and control cells (Fig. 4a). Whole-transcriptome analysis identified 209 upregulated genes and 267 downregulated genes; however, particular attention was paid to the TRIM11-regulated genes, which are related to cancer drug resistance and showed a >2-fold change in the TRIM11-KO group vs the control group in the RNA sequencing results. Subsequent validation by qRT-PCR and western blotting revealed that the ABCC9 expression levels were consistently decreased in the TRIM11-KO groups compared with those in the control groups (Fig. 4b, c) and were increased in the TRIM11 overexpression groups compared with those in the control groups (Fig. 4b, c). Numerous studies have suggested that upregulation of the ATP-binding cassette (ABC) family proteins promotes drug resistance in various cancers by mediating the efflux of cytotoxic chemotherapeutics^28^; ABCC9 belongs to the ABCC subfamily, which is best known for containing the majority of drug transporters and multidrug resistance proteins (MRPs)^28^. Using the Oncomine online database, we found that the ABCC9 expression levels were significantly higher in NPC tissues than in normal nasopharyngeal epithelial tissues (Supplementary Fig. 5). Interestingly, qRT-PCR and western blotting were performed and showed that the mRNA and protein levels of ABCC9 increased in CNE1-DDP and CNE2-DDP cells compared with those in CNE1 and CNE2 cells, respectively (Fig. 4d). In addition, the real-time PCR results showed that ABCC9 was significantly increased in secondary tissues (Fig. 4e), and a significant positive correlation was identified between TRIM11 and ABCC9 in tissues (Fig. 4f).

The above results indicate that ABCC9 may play an oncogenic role in NPC. We thus analyzed the contribution of ABCC9 to TRIM11-induced drug resistance. As expected, knockdown of ABCC9 by siRNA abolished TRIM11-mediated drug resistance (Fig. 4g–k). These results suggest that the ability of TRIM11 to promote drug resistance is strictly dependent on the upregulation of ABCC9.

### Result 5. TRIM11 activated Wnt/β-catenin signaling to induce ABCC9

**Fig. 5:**
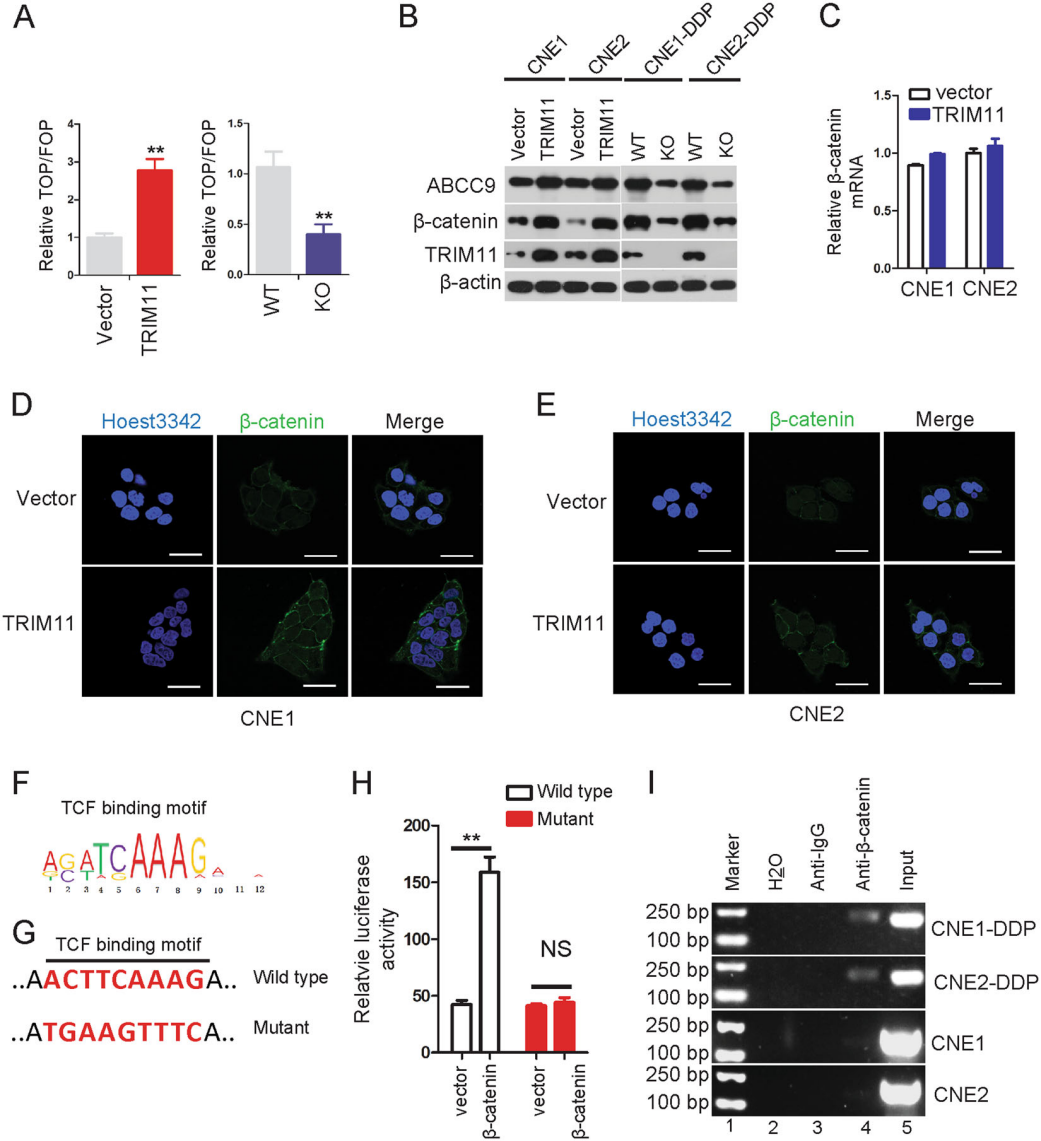
TRIM11 promoted the protein level of β-catenin to directly upregulate ABCC9. **a** Overexpression of TRIM11 significantly increased TOPflash/FOPflash luciferase reporter activity in CNE2 cells, while KO of TRIM11 significantly suppressed Wnt activity in CNE2-DDP cells. **P < 0.01. **b** Western blot analysis results showed that TRIM11 expression enhanced the expression of ABCC9 and β-catenin, while KO of TRIM11 showed the opposite effect. **c** The mRNA levels of β-catenin were detected after overexpressing TRIM11 in CNE1 and CNE2 cells. **d**, **e** The protein level of β-catenin increased in the cytoplasm and nucleus, as demonstrated by confocal immunofluorescence analysis in CNE1 and CNE2 cells after stably overexpressing TRIM11. Scale bar, 20 µm. **f** The TCF/LEF motif is shown. **g** Schematic illustration of the WT TCF/LEF motif sequences within the ABCC9 promoter and its mutants for luciferase reporter assays. **h** CNE2 cells stably transfected with an empty vector or TRIM11-encoding plasmid were transfected with a luciferase reporter plasmid in which luciferase expression was driven by a WT or mutant ABCC9 promoter. Luciferase activity was measured as described in the “Materials and methods” section. Data are the mean ± SD of triplicate samples. **P < 0.01. **i** Cells were analyzed in ChIP assays using anti-β-catenin antibody as described in the “Materials and methods” section.

To precisely identify the molecular mechanisms underlying TRIM11-induced ABCC9 expression in NPC, we performed luciferase reporter assays to assess the effect of TRIM11 on important cancer pathway. Ectopic expression of TRIM11 significantly increased the activity of Wnt signaling, as demonstrated by a TOPflash/FOPflash luciferase reporter assay, and KO of TRIM11 showed the opposite effect on Wnt activity (Fig. 5a). Indeed, overexpression of TRIM11 significantly upregulated the β-catenin protein level, while TRIM11 KO diminished β-catenin expression (Fig. 5b). However, qRT-PCR showed that the β-catenin mRNA abundance was not altered with TRIM11 overexpression in CNE1 and CNE2 cells (Fig. 5c). Using immunofluorescence to visualize intracellular β‐catenin, we observed an increase in the β‐catenin levels and the translocation of β‐catenin from the cell membrane to the nucleus, which is the key step for its transcriptional activation (Fig. 5d, e).

To elucidate the mechanisms underlying β-catenin-induced upregulation of ABCC9, we analyzed the ABCC9 promoter and identified a TCF-binding motif as potential binding sites for β-catenin (Fig. 5f) and then constructed luciferase reporter vectors with a WT promoter or TCF-binding site mutant promoters (Fig. 5g). Luciferase assays showed that β-catenin upregulated the WT luciferase activity but not the mutant luciferase activity (Fig. 5h), indicating that the TCF-binding site was the key region for β-catenin-mediated upregulation of ABCC9. A ChIP assay was performed to analyze whether β-catenin directly binds to the above mentioned region. The results indicate that β-catenin directly binds to the ABCC9 promoter in NPC cells (Fig. 5i), and this binding was greater in the cell lines CNE1-DDP and CNE2-DDP than in cell lines CNE1 and CNE2 (Fig. 5i). The negative control assay was performed and showed that β-catenin did not bind to the area beyond the β-catenin binding region in CNE1-DDP cells (Supplementary Fig. 6).

### Result 6. TRIM11 facilitates the degradation of Daple by inducing ubiquitination

**Fig. 6:**
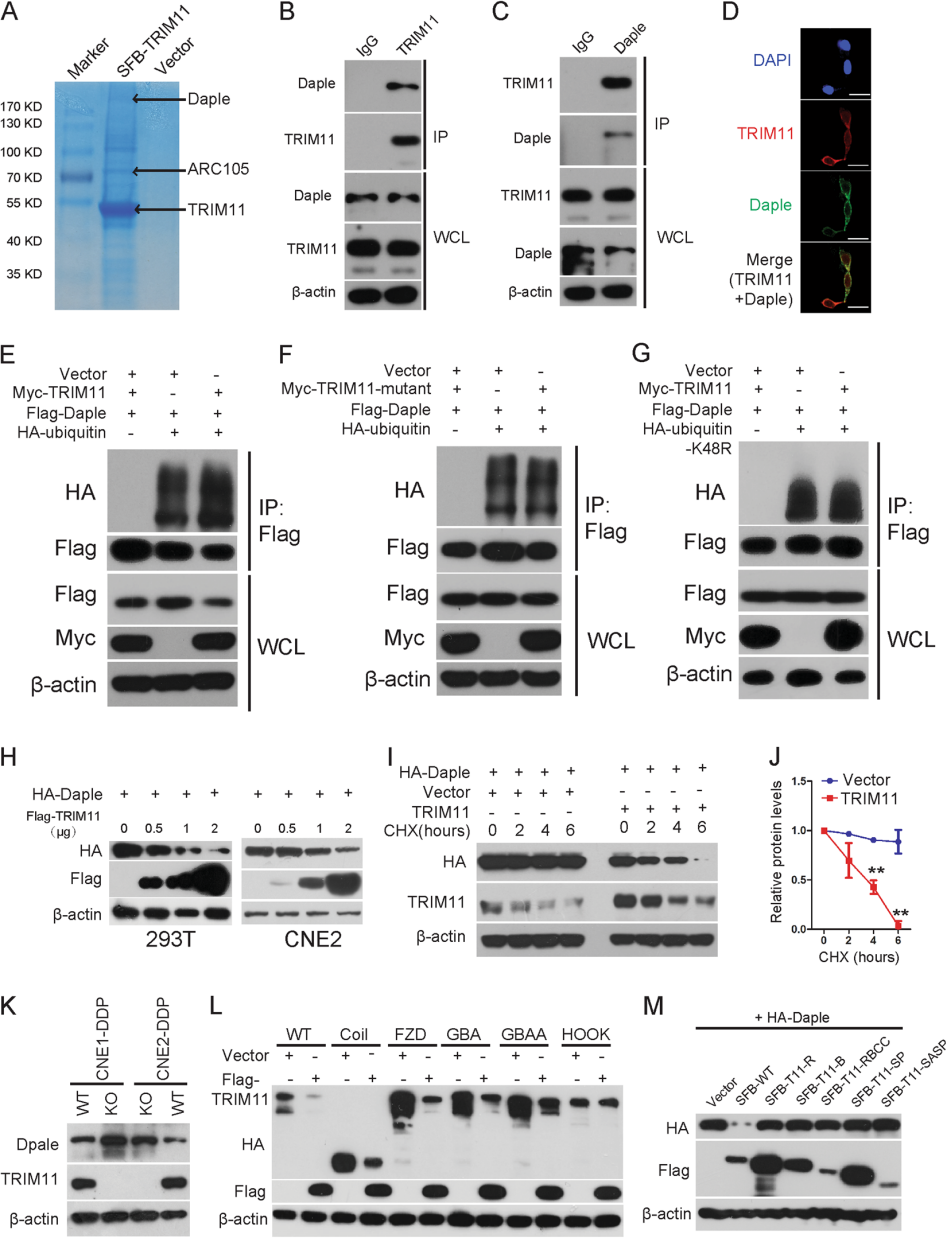
TRIM11 facilitates the degradation of Daple by inducing ubiquitination. **a** CNE2 cells stably expressing SFB-TRIM11; whole-cell lysates were obtained by tandem affinity purification of the TRIM11-associated protein complex as described in the “Materials and methods” section, and affinity-purified TRIM11 complexes were visualized by Coomassie brilliant blue staining. TRIM11 and Daple protein bands are indicated by arrows. **b**, **c** CNE2-DDP cells were harvested for immunoprecipitation using anti-IgG, anti-TRIM11 or anti-Daple antibodies and then analyzed by western blotting. β-actin was used as a loading control. **d** CNE2-DDP cells were fixed after cotransfection with Flag-TRIM11 and HA-Daple for 24 h and analyzed by immunofluorescence analysis. Scale bar, 20 µm. **e**, **f** Determination of WT TRIM11-mediated (**e**) or mutant TRIM11-mediated (**f**) ubiquitinated Daple. Lysates from cells transfected with the indicated constructs plus HA-tagged ubiquitin were immunoprecipitated with S beads, and the immunoprecipitates were analyzed by western blotting using an anti-HA antibody to detect the Daple/ubiquitin complexes. **g** Determination of the levels of ubiquitinated Daple between WT and mutant K48R ubiquitin. Lysates from cells transfected with the indicated constructs plus HA-tagged ubiquitin were immunoprecipitated with S beads, and the immunoprecipitates were analyzed by western blotting using an anti-HA antibody to detect the Daple/ubiquitin complexes. **h** Immunoblot analysis of protein extracts of CNE2 or 293T cells transfected with plasmid for HA-Daple and increasing doses of plasmid for Flag-TRIM11. β-actin was used as a loading control. **i**, **j** CNE2 cells stably expressing empty vector or TRIM11 were incubated with 20 μg/ml CHX for the indicated times. Cell lysates were then analyzed by western blotting (**i**), and the densities of the HA-Daple protein bands at each time point were normalized to β-actin and were calculated as percentages (**j**). Data are the mean ± SD of triplicate samples. **P < 0.01. **k** The endogenous protein level of Daple was detected after knocking out TRIM11 in CNE1-DDP and CNE2-DDP cells. β-actin was used as a loading control. **l** Immunoblot analysis of extracts of CNE2 cells transfected with plasmids for HA-Daple or its mutants, together with Flag-TRIM11. β-actin was used as a loading control. **m** Immunoblot analysis of extracts of CNE2 cells transfected with plasmids for SFB-TRIM11 or its mutants, together with HA-Daple. β-actin was used as a loading control.

Since TRIM11 is an E3 ubiquitin ligase that usually interacts with target proteins to regulate the cell phenotype, we performed tandem affinity purification to identify the key downstream targets of TRIM11 using CNE2 cells stably expressing triple epitope (S-peptide, FLAG, and streptavidin-binding peptide)-tagged TRIM11 (SFB-TRIM11) (Fig. 6a). Mass spectrometry analysis revealed that in addition to ARC105, which was reported to associate with TRIM11 to regulate gene transcription^29^, Daple is a potential TRIM11-associated protein and was identified as an inhibitor of Wnt/β-catenin signaling by binding to Dvl^16^ (Fig. 6a; Supplementary Table S4). Indeed, knockdown of Daple by CRISPR/Cas9 gene editing in CNE2-DDP enhanced β-catenin and ABCC9 expression (Supplementary Fig. 7a), while overexpression of Daple reduced β-catenin and ABCC9 expression and reversed TRIM11-mediated upregulation of β-catenin and ABCC9 expression (Supplementary Fig. 7b). Moreover, Daple also reduced and suppressed TRIM11-mediated cell viability and colony formation abilities under DDP exposure in vitro (Supplementary Fig. 7c–e). An in vivo tumorigenesis study was also performed by inoculating CNE2-DDP-NC cells or CNE2-DDP-Daple knockdown cells into nude mice. Mice treated with DDP or saline were sacrificed 2 weeks after inoculation, and the results showed that the Daple knockdown group exhibited significantly reduced drug sensitivity compared with that in the NC group (Supplementary Fig. 7f, g). These results suggest that the Daple/β-cantenin/ABCC9 axis is a crucial TRIM11 downstream regulator for drug resistance in NPC.

To further confirm this interaction, following coimmunoprecipitation (co-IP) using HA-agarose or S beads, a complex containing TRIM11 and Daple was clearly detected in CNE2 cells overexpressing both exogenous SFB-tagged TRIM11 and HA-tagged Daple (Supplementary Fig. 8a, b). More importantly, the complex containing TRIM11 and Daple was detectable at their endogenous levels in CNE2-DDP cells, as shown in Fig. 6b, c. We also examined the subcellular localization of TRIM11 and Daple by immunofluorescence, as shown in Fig. 6d. SFB-TRIM11 colocalized extensively with HA-Daple in the cytoplasm. Series of deletions of TRIM11 and Daple were generated, and coimmunoprecipitation was performed to map the binding domains of the two proteins. As shown in Supplementary Fig. 8c, the coiled-coil, SPRY-associated and SPRY domains of TRIM11 are important for its binding to Daple. The hook domain of Daple contributed to the interaction with TRIM11 (Supplementary Fig. 8d). These results reinforce the hypothesis that TRIM11 physically and specifically interacts with Daple in cells.

TRIM11 can promote the degradation of target proteins via the ubiquitin-proteasome or autophagosome system^30–32^. Thus, we used an in vivo ubiquitination assay to test whether TRIM11 promotes Daple ubiquitination. 293T cells transfected with Flag-Daple and HA-ubiquitin in the absence or presence of myc-TRIM11 were treated with MG132 and Baf-A1 for 3 h to stabilize the ubiquitinated proteins before lysis. In the absence of ectopic myc-TRIM11, Daple was weakly ubiquitinated, whereas cotransfection of myc-TRIM11 increased the ubiquitinated Daple level (Fig. 6e). Moreover, deletion of either SPRY domain abolished TRIM11-induced Daple ubiquitination (Fig. 6f). The formation of lysine 48-linked polyubiquitination chains is the principal signal for proteolysis. To clarify which polyubiquitination chain mediates this ubiquitination, 293T cells were transfected with Flag-Daple, myc-TRIM11 and/or ubiquitin K48R, as indicated. In the case of cotransfection of TRIM11, TRIM11-mediated ubiquitination of Daple was exclusively dependent on lysine 48-linked polyubiquitin chains (Fig. 6g). These results indicate that TRIM11 facilitates the ubiquitination of Daple via lysine 48-linked polyubiquitin chains.

Next, based on the observation that TRIM11 targets Daple for ubiquitination, we detected whether TRIM11 promoted Daple turnover. HA-Daple was cotransfected with different amounts of SFB-TRIM11 into 293T and CNE2 cells. Indeed, TRIM11 overexpression reduced Daple protein levels in a dose-dependent manner (Fig. 6h); ectopic expression of SFB-TRIM11 notably reduced the half-life of Daple using the CHX chase assay (Fig. 6i, j). Knocking out TRIM11 in CNE1-DDP and CNE2-DDP increased the levels of the endogenous Daple protein (Fig. 6k), which suggested that KO of TRIM11 enhanced β-catenin degradation by Daple-mediated inhibition of Dvl. Moreover, mutation of the HOOK domain of Daple abrogated its degradation by TRIM11 (Fig. 6l). Deletion of any domain of TRIM11 abolished TRIM11-mediated Daple turnover (Fig. 6m), which indicated that intact TRIM11 is essential for E3 ligase activity. Taken together, these results indicate that TRIM11 functions as an E3 ubiquitination ligase to mediate Daple degradation.

### Result 7. TRIM11 mediates selective autophagic degradation of Daple

**Fig. 7:**
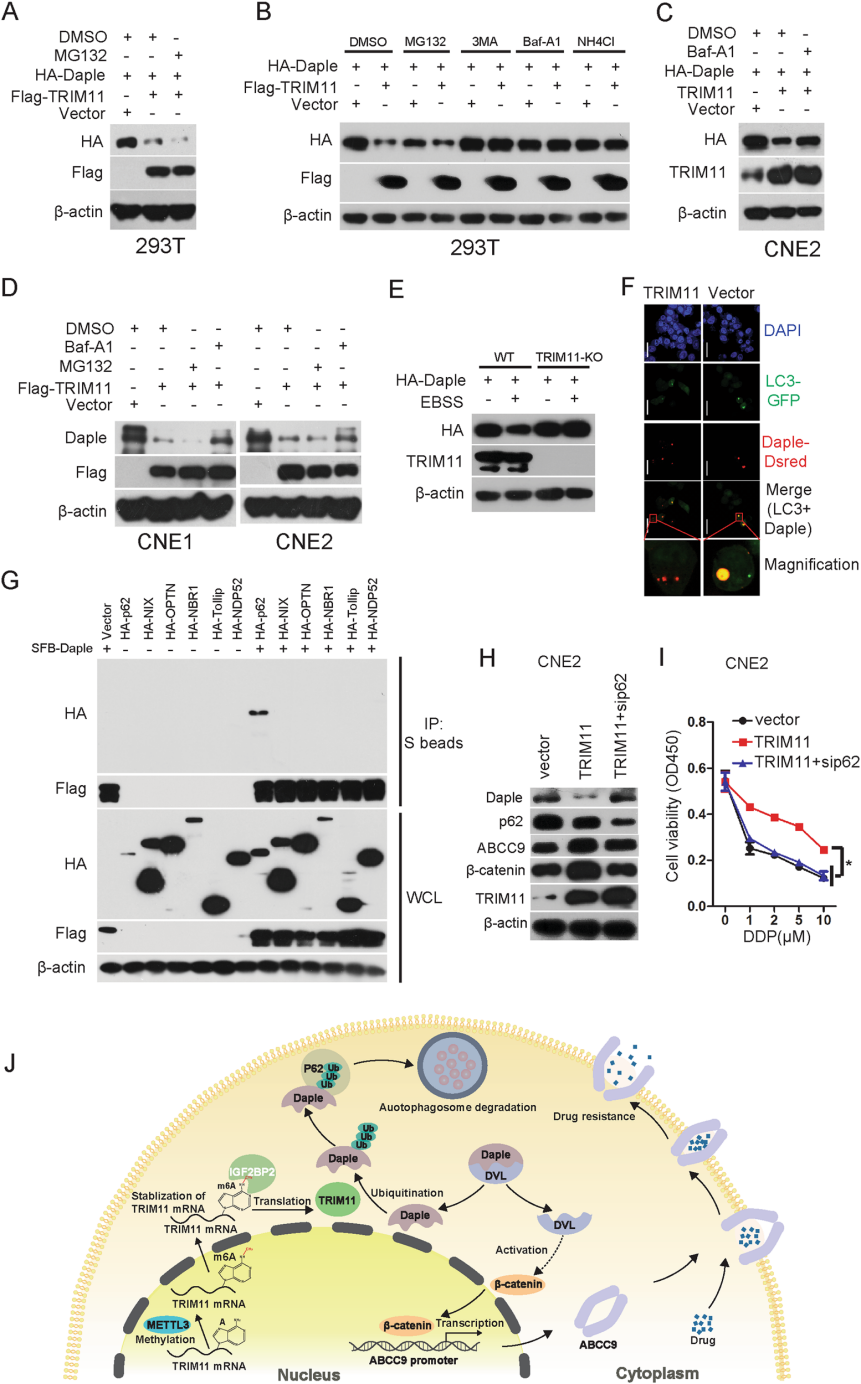
TRIM11 mediates selective autophagic degradation of Daple. **a** Immunoblot analysis of extracts of 293T cells transfected with plasmids for Flag-TRIM11 and HA-Daple and treated with DMSO or MG-132 (10 mM) for 6 h. β-actin was used as a loading control. **b** Immunoblot analysis of extracts of 293T cells transfected with plasmids for Flag-TRIM11 and HA-Daple and treated with DMSO (vehicle) or MG-132 (10 mM), 3-MA (10 mM), Baf-A1 (1 μM), or NH_4_Cl (20 mM) for 6 h. **c** Immunoblot analysis of extracts of CNE2 cells transfected with plasmids for Flag-TRIM11 and HA-Daple and treated with DMSO (vehicle) or Baf-A1 (1 μM) for 6 h. β-actin was used as a loading control. **d** The endogenous protein level of Daple was detected in CNE1 and CNE2 cells transfected with plasmids for Flag-TRIM11 and treated with DMSO (vehicle), MG-132 (10 mM), or Baf-A1 (1 μM) for 6 h. β-actin was used as a loading control. **e** WT and TRIM11-KO CNE2-DDP cells were transfected with HA-Daple for 20 h and treated with EBSS for 4 h. Cell lysates were then collected for western blotting. **f** The indicated cells transfected with GFP-LC3B and DsRed-Daple were challenged with CQ (50 mM) for 6 h. Subsequently, the cells were subjected to immunofluorescence staining and confocal microscopy analysis. Scale bar, 20 µm. **g** Co-IP and immunoblot analysis of 293T SFB-Daple cells cotransfected with empty vector for certain autophagic components, including HA-tagged p62, NIX, OPTN, NBR1, Tollip, and NDP52. **h** The protein levels of ABCC9, TRIM11, p62, β-catenin, and Daple were determined in the indicated cells. **i** The indicated cells were treated with the indicated concentrations of DDP for 24 h, and cell viability was assessed with a CCK-8 assay. The bars correspond to the mean ± standard error (*n* = 3), and the *P* value was calculated using Student’s *t* test. *P < 0.05. **j** Working model of m6A-mediated upregulation of TRIM11 facilitates chemoresistance in NPC by activating the β-catenin/ABCC9 axis via p62-selective autophagic degradation of Daple.

Usually, there are three systems of protein degradation in cells, namely, the proteasome, lysosome, and autolysosome pathways. To determine the pathway by which TRIM11 promotes the degradation of Daple, we treated TRIM11-overexpressing cells with the proteasome inhibitor MG132, but the protein level of Daple was not rescued (Fig. 7a); however, the protein level of Daple was rescued after TRIM11-overexpressing cells were treated with the autophagy inhibitors CQ, Baf-A1 or 3-methyladenine (3-MA), as well as the lysosomal inhibitor NH_4_Cl (Fig. 7b, c). We also found that the endogenous protein level of Daple was rescued after treatment with Baf-A1 but not after treatment with MG132 (Fig. 7d). Next, we found that the degradation of Daple induced by EBSS was impaired in TRIM11-KO cells (Fig. 7e), suggesting that TRIM11 plays a critical role in mediating autophagy-dependent degradation of Daple. The colocalization of Daple and LC3 was examined by cotransfection of GFP-LC3 and DsRed-Daple into 293T cells after transfection with a vector or TRIM11 and treatment with the autophagy inhibitor Baf-A1. As expected, the colocalization of Daple and LC3 was clearly observed in the autolysosome when the two confocal images were merged (Fig. 7f). Moreover, the degradation of Daple mediated by TRIM11 was totally blocked in BECN1-KO and LC3B-KO cells (Supplementary Fig. 9a, b). These results suggest that TRIM11 mediates Daple degradation though the autolysosome pathway.

Recent work has identified mechanisms for the selective uptake of cytoplasmic proteins into nascent autophagic vesicles^33^. Selectivity in autophagy is achieved through cargo recognition by a family of autophagy receptors^33^. Using co-IP assays, we found that Daple specifically interacted with p62 but not with other cargo receptors, including NIX, OPTN, NRB1, tollip or NDP52 (Fig. 7g). Moreover, we found that Daple did not interact with other proteins involved in autophagy initiation and elongation, including members of the beclin-1 complex or the ULK1 complex, ATG5-12, or ATG7 (Supplementary Fig. 9c). Importantly, knockdown of p62 by siRNA could rescue Daple protein level in TRIM11 overexpression cells (Fig. 7h). Accumulating evidence has indicated that high levels of p62 are implicated in causing resistance to cancer therapy^34^. Indeed, knockdown of p62 suppressed TRIM11-mediated DDP resistance (Fig. 7i). The above results show that Daple is recruited to the autophagosome during its maturation and that p62 is critical for the TRIM11-mediated selective autophagic degradation of Daple to promote chemoresistance.

## Discussion

Approximately, 80 TRIM genes have been identified in humans and are associated with a variety of physiological processes, including cell proliferation, DNA repair, drug resistance, pluripotency, signal transduction, and transcription^9,35,36^. Alterations in TRIM proteins are thought to be involved in various pathological conditions, including cancer; however, the oncogenic or tumor suppressor function of TRIMs is context dependent^36^. The major finding in this work is the discovery of TRIM11-mediated drug resistance of NPC, which targeted Daple for its degradation via p62-selective autophagy to activate β-catenin/ABCC9 signals (Fig. 7j).

Di et al.^37^ first reported that TRIM11 functions in an oncogenic role to promote proliferation, invasion, migration, and glioma tumor growth by improving the accumulation of EGFR and the activity of the MAPK cascade in malignant gliomas. Subsequently, TRIM11 was also shown to exert an oncogenic role in lung cancer, liver cancer, breast cancer, and ovarian cancer via various mechanisms^38–41^. We previously reported that TRIM11 was expressed at higher levels in clinical colon cancer tissues than in adjacent noncancerous tissues, and higher TRIM11 mRNA levels predicted a poor outcome. We also found that TRIM11 could promote colon cancer cell proliferation and colony formation, inhibit apoptosis in vitro, and promote colon tumor growth in vivo^21^. However, the role of TRIM11 in the regulation of tumor drug resistance remains unknown. Here, we defined a critical role of TRIM11 in the positive regulation of NPC chemoresistance.

The Dvl protein, a key component of Wnt signaling, relays Wnt signals from receptors to downstream effectors^42^. In the canonical Wnt pathway, Dvl is recruited by the receptor Frizzled and prevents the constitutive destruction of cytosolic β-catenin^42^. Daple inhibits canonical Wnt signals by forming Daple–Dvl complexes. Daple stood out as a strong candidate to explore the molecular mechanism by which TRIM11 upregulated β-catenin after utilizing Co-IP/LC–MS/MS for the identification of direct targets of TRIM11. Our results show that TRIM11 promotes ubiquitination and degradation of Daple through its E3 ligase activity. TRIM11 promotes substrate degradation through proteasome and autophagy–lysosome pathways^31,43^. Our results showed that TRIM11 mediates p62-selective autophagic degradation to induce β-catenin accumulation. Chen et al. confirmed that inhibiting p62 enhanced sensitivity to cisplatin in NPC cells^44^, our data present new evidence as well as a regulatory mechanism for p62‐mediated drug resistance in NPC.

The ABC transporters utilize the energy stored in adenosine triphosphate (ATP) to translocate specific substrates such as chemotherapeutic drugs across the membrane or to regulate the activity of membrane channels^45^. ABC exporters that can extracellularly transport chemotherapeutic drugs have been linked with multidrug resistance in both bacterial and eukaryotic cells^46^. Members of the ABCC subfamily are known as MRPs and are overexpressed in cancers, where they contribute to cancer chemoresistance and treatment failure^28^. Our results indicate that ABCC9, a member of the ABCC subfamily, is overexpressed in NPC drug-resistant cells and is directly regulated by the TRIM11/Daple/β-catenin axis, which explains why TRIM11 promotes drug resistance in NPC.

There are more than 100 different chemical modification reactions of RNA, among which methylation is the most widely studied^47^, and m6A is the most abundant internal modification in the transcriptome with potentially important roles in mRNA metabolism and multiple biological processes^47^. Aberrant m6A modification participates in promoting cancer chemoresistance via deregulation of downstream signaling pathways^48,49^. Here, we found that the m6A level of TRIM11 is higher in CNE1-DDP and CNE2-DDP cells than in CNE1 and CNE2 cells, and m6A modification of TRIM11 mRNA improves its RNA stability, which may at least partially account for the upregulation of TRIM11 in NPC drug-resistant cells. Our results also report for the first time that METTL3 promotes drug resistance in NPC, at least partially by regulating the expression of TRIM11.

In conclusion, our present study has highlighted the importance of TRIM11 in promoting the drug resistance of NPC by regulating Daple-Dvl-β-catenin-ABCC9 signaling. Hence, a therapeutic intervention that interrupts the functional interplay between TRIM11 and Daple might provide a promising strategy to treat NPC.

### Availability of data and materials

All data generated or analyzed during this study are included in this published article and its Additional files.

### Supplementary information

Supplementary Figures

Supplementary Table 1

Supplementary Tables 2 and 3
