# Correction: TRIM11 facilitates chemoresistance in nasopharyngeal carcinoma by activating the β-catenin/ABCC9 axis via p62-selective autophagic degradation of Daple

**DOI:** 10.1038/s41389-023-00506-x

**Published:** 2024-01-10

**Authors:** Runa Zhang, Si-Wei Li, Lijuan Liu, Jun Yang, Guofu Huang, Yi Sang

**Affiliations:** 1 Jiangxi Key Laboratory of Cancer Metastasis and Precision Treatment, The Third Affiliated Hospital of Nanchang University, Nanchang, People’s Republic of China; 2 Department of Oncology, Tongji Huangzhou Hospital of Huazhong University of Science and Technology, Hubei, People’s Republic of China; 3 Department of Pharmacy, Jiangxi Cancer Hospital, Nanchang, Jiangxi 330029 People’s Republic of China

**Keywords:** Oncogenes, Cell signalling

Correction to: *Oncogenesis* 10.1038/s41389-020-0229-9, published online 07 May 2020

Following the publication of this study, the authors noted the omission of grant number 20204BCJL23052 (Jiangxi Provincial Natural Science Foundation of China) from the acknowledgments section. This has now been added.

The original article has been corrected.

