# Supplementary material for: TRIM11 facilitates chemoresistance in nasopharyngeal carcinoma by activating the β-catenin/ABCC9 axis via p62-selective autophagic degradation of Daple
Source: Oncogenesis. 2020 May 7;9(5):45. doi: 10.1038/s41389-020-0229-9 (PMC7206012; doi:10.1038/s41389-020-0229-9)
Supplement: Supplementary file 1 — Supplementary Figures [file 41389_2020_229_MOESM1_ESM.docx]

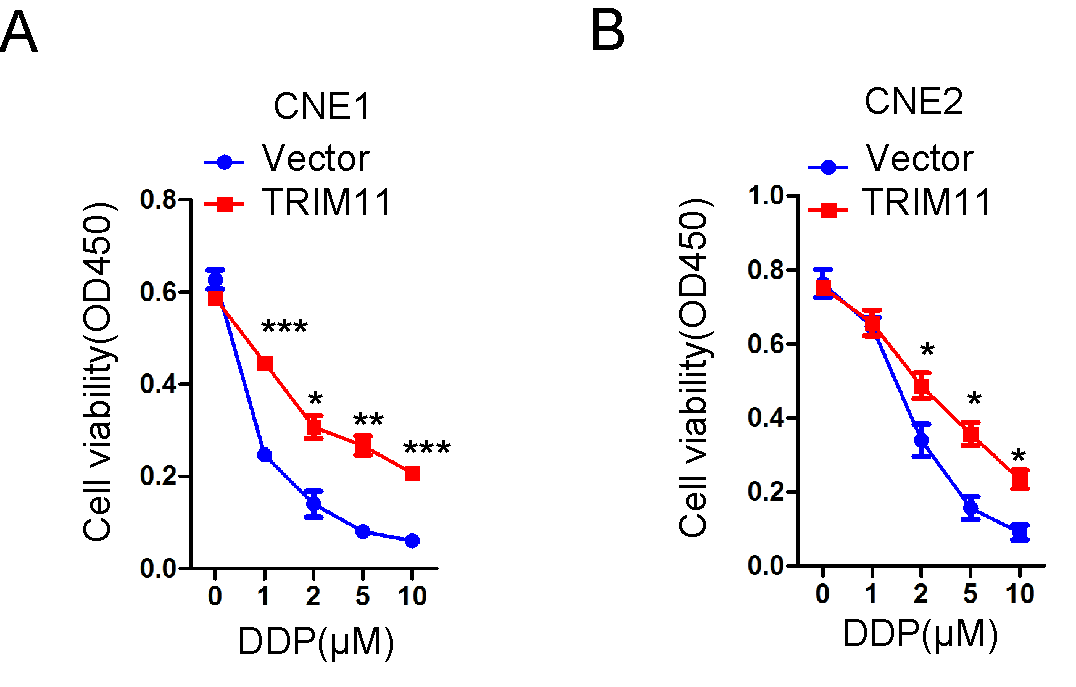


**Supplementary Figure 1. METTL3 strengthens chemoresistance to DDP.** (A, B) The indicated cells were treated with the indicated concentrations of DDP for 24 h, and cell viability was assessed with the CCK-8 assay. The bars correspond to the mean ± standard error (n = 3), and the P value was calculated using Student’s t-test. *P < 0.05.**P < 0.01. ***P < 0.001.


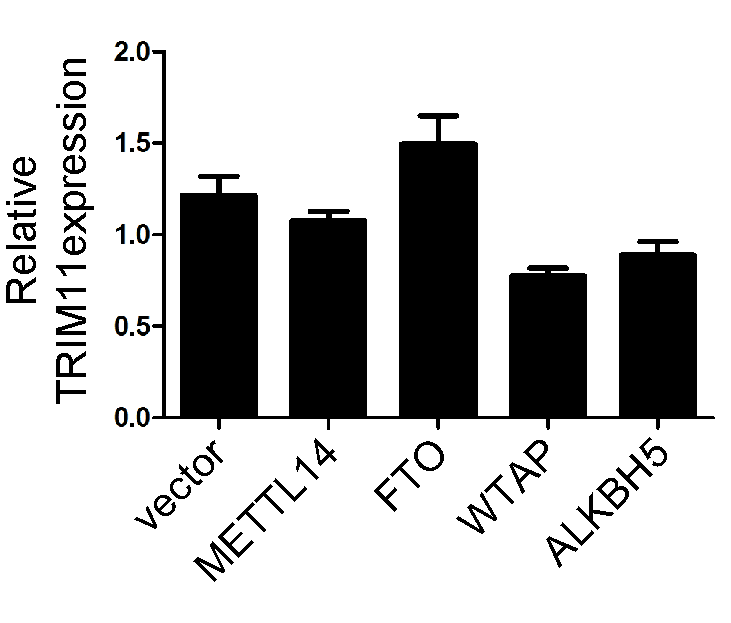


**Supplementary Figure 2.** The mRNA level of TRIM11 was detected after overexpression of METTL14, WTAP, FTO or ALKBH5 in CNE2 cells. GAPDH was used as a loading control.


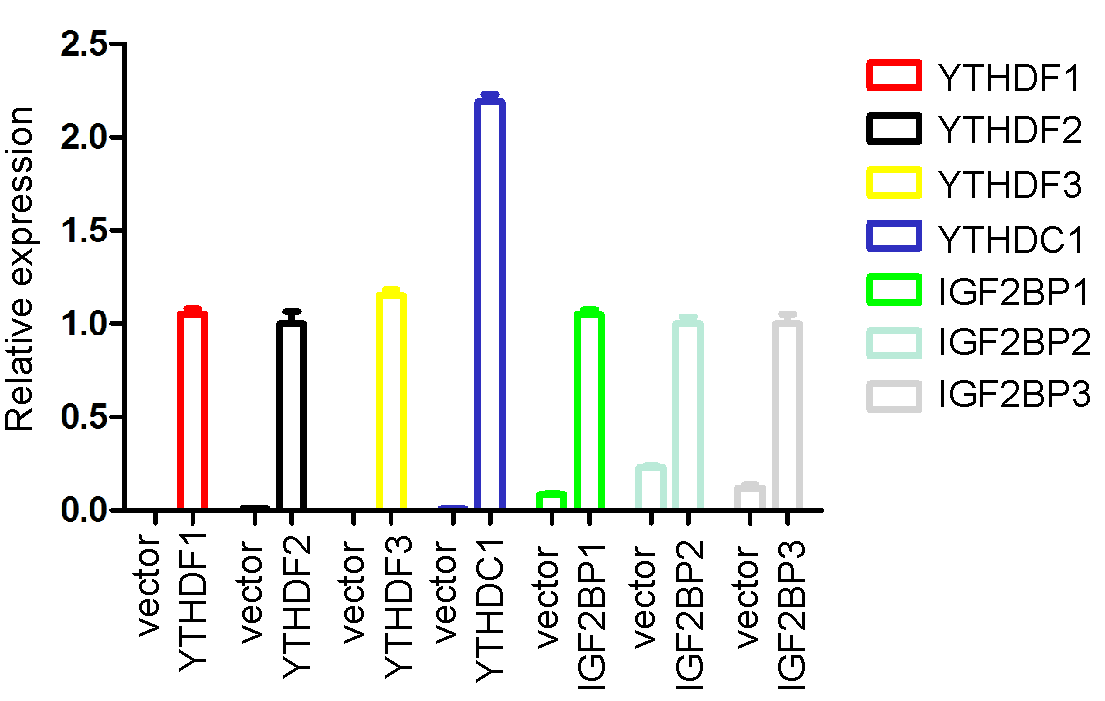


**Supplementary Figure 3.** The genes expression were detected after overexpression of YTHDF1, YTHDF2, YTHDF3, YTHDC1, IGF2BP1, IGF2BP2 or IGF2BP3 in CNE2 cells. GAPDH was used as a loading control.


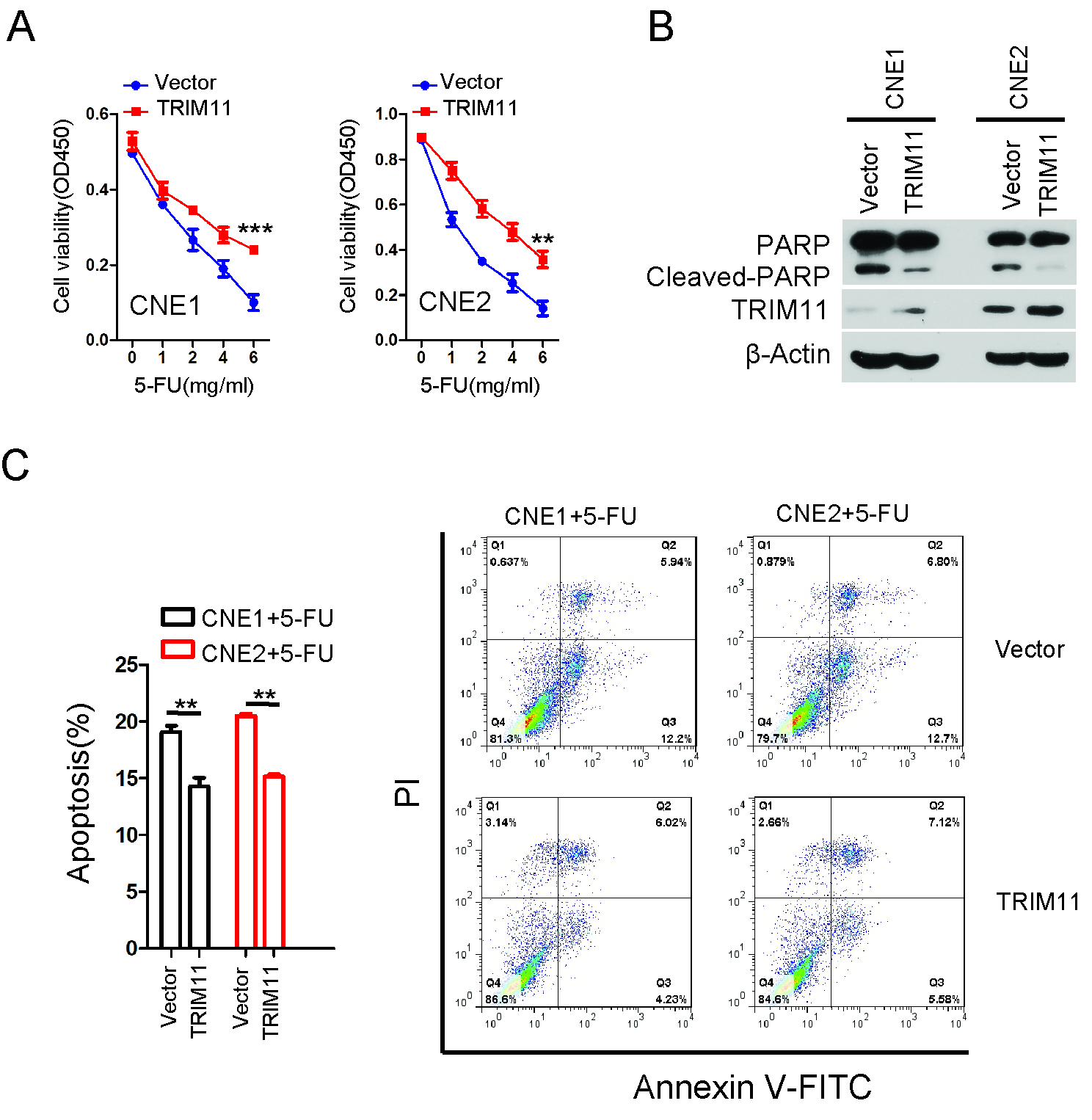


**Supplementary Figure 4. TRIM11 strengthens chemoresistance to 5-FU and docetaxel in vitro.** (A) The indicated cells were treated with the indicated concentrations of 5-FU for 24 h, and cell viability was assessed with the CCK-8 assay. The bars correspond to the mean ± standard error (n = 3), and the P value was calculated using Student’s t-test. (B) The indicated stable cells were treated with 5-FU (2mg/ml) for 24 h and subjected to western blotting. (C) The indicated stable cell lines were treated with 5-FU (2mg/ml) for 24 h and then subjected to annexin V-FITC and PI staining. Cell apoptosis was evaluated through FACS. The bars correspond to the mean ± standard error (n = 3), and the P value was calculated using Student’s t-test. **P < 0.01.


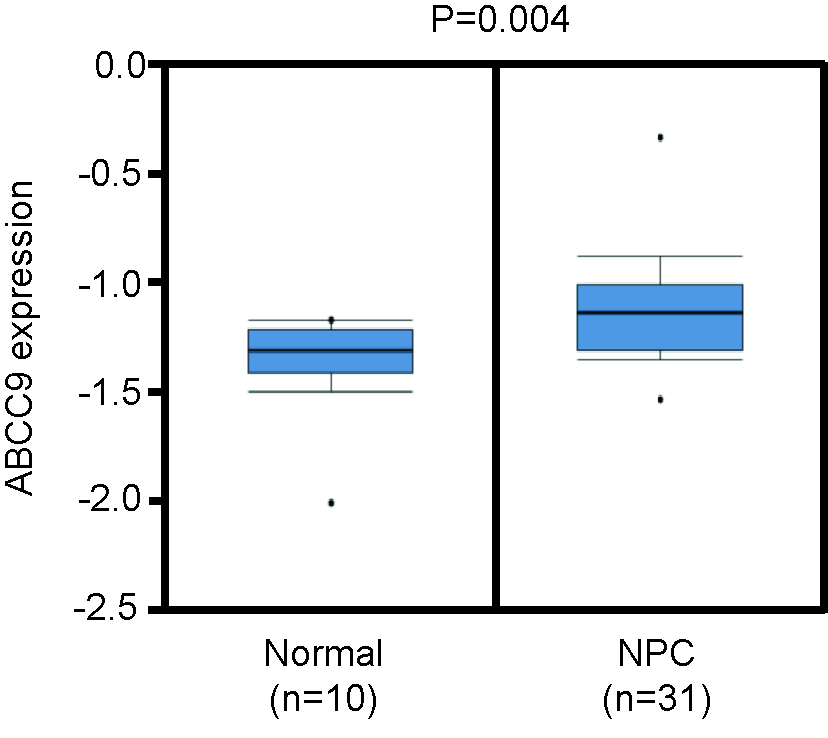


**Supplementary Figure 5.** Meta-analysis of ABCC9 mRNA levels in NPC samples from the Oncomine database (http://www.oncomine.org).


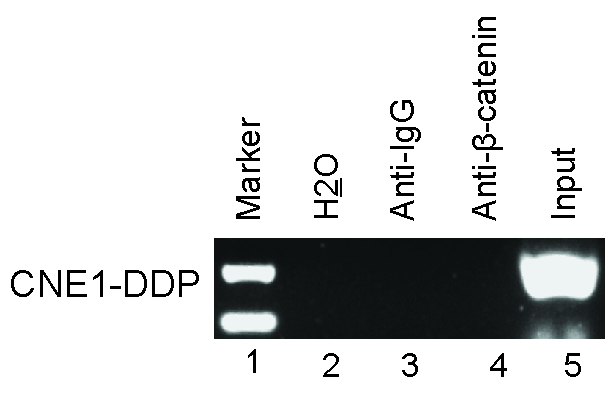


**Supplementary Figure 6.** Cells were analyzed in ChIP assays using anti-β-catenin antibody as described in the Methods section.


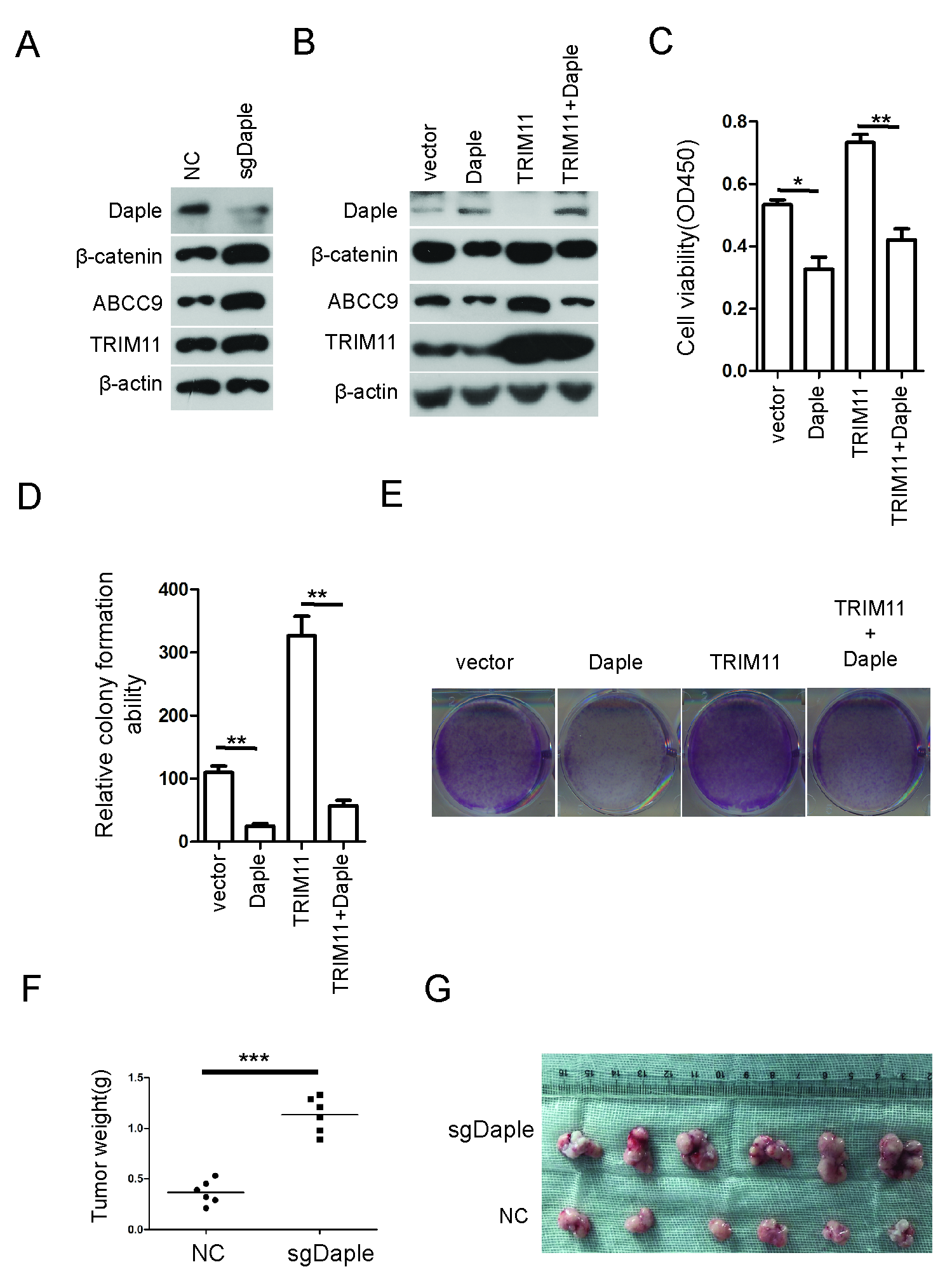


**Supplementary Figure 7.** **Daple suppressed chemoresistance in NPC** (A) The protein levels of ABCC9, TRIM11, β-catenin and Daple were determined after knocking down Daple in CNE2-DDP cells. (B) The protein levels of ABCC9, TRIM11, β-catenin and Daple were determined. (C) The indicated cells were treated with the indicated concentrations of DDP for 24 h, and cell viability was assessed with a CCK-8 assay. The bars correspond to the mean ± standard error (n = 3), and the P value was calculated using Student’s t-test. (D, E) The colony formation of the indicated stable cell lines in vitro was measured, as described in the Methods section. E is the representative images. (F，G) A xenograft model consisting of nude mice with CNE2-DDP-NC and CNE2-DDP-sgDaple cells was injected into the armpits of 4-week-old mice (n = 6/group). Images of tumors from the mice (G). Mean tumor weights were calculated (F). The results are presented as the means ± SDs. ***P < 0.001.


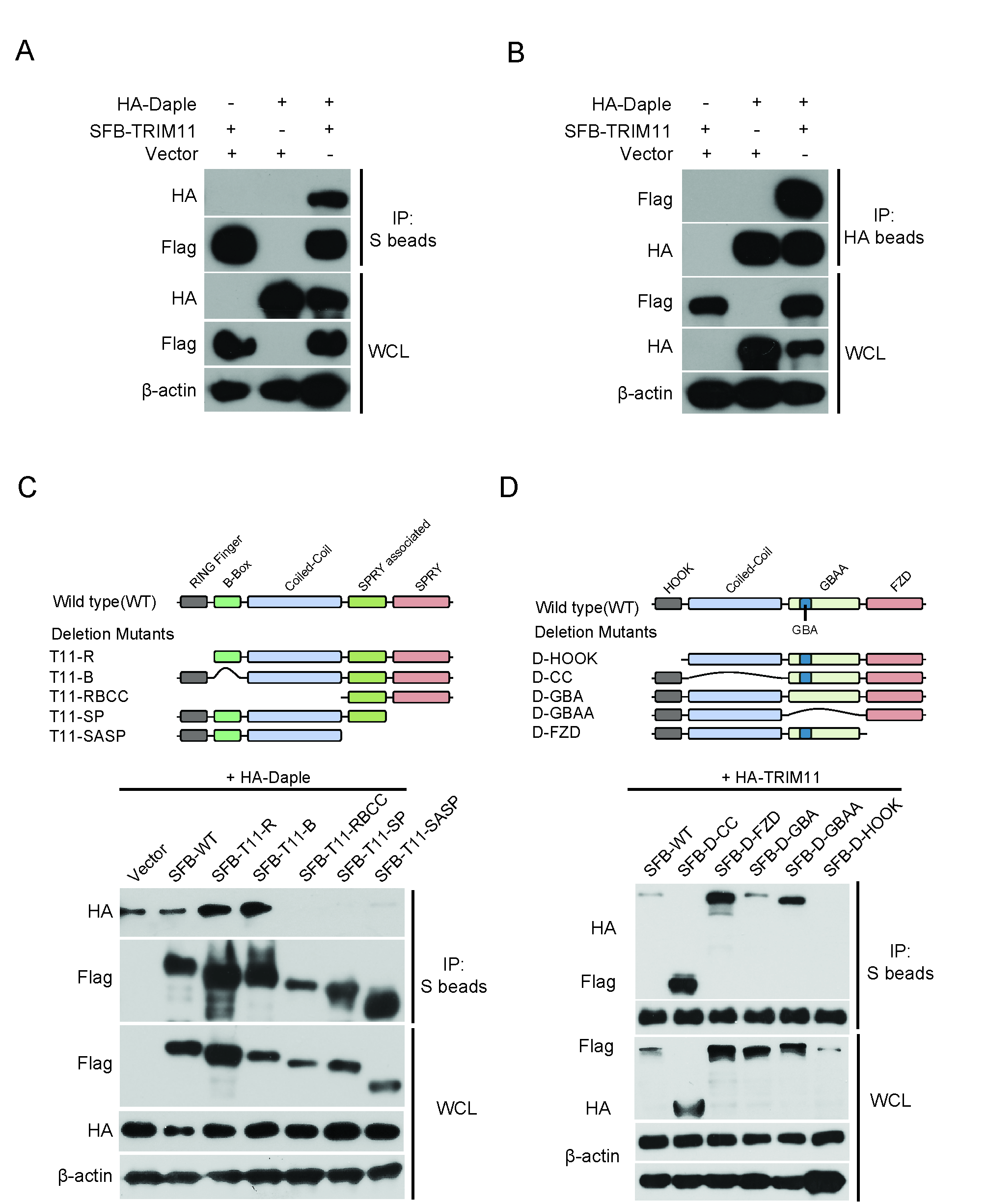


**Supplementary Figure 8.** **TRIM11 interacted with Daple.** (A, B) CNE2 cells transfected with SFB-TRIM11 or HA-Daple plasmids for 24 h and lysed with RIPA buffer. S beads (A) or HA-agarose (B) were used for immunoprecipitation (IP) and western blotting with the indicated antibodies. β-actin was used as a loading control. (C) Top: the domain structure of TRIM11. Bottom: CNE2 cells were cotransfected with HA-Daple and SFB-TRIM11 or SFB-TRIM11 deletion mutants. S beads were used for immunoprecipitation (IP) and western blotting with the indicated antibodies. β-actin was used as a loading control. (D) Top: the domain structure of Daple. Bottom: CNE2 cells were transfected with SFB-Daple and HA-TRIM11 or SFB-Daple deletion mutants. S beads were used for immunoprecipitation (IP), and western blotting with the indicated antibodies was performed. β-actin was used as a loading control.


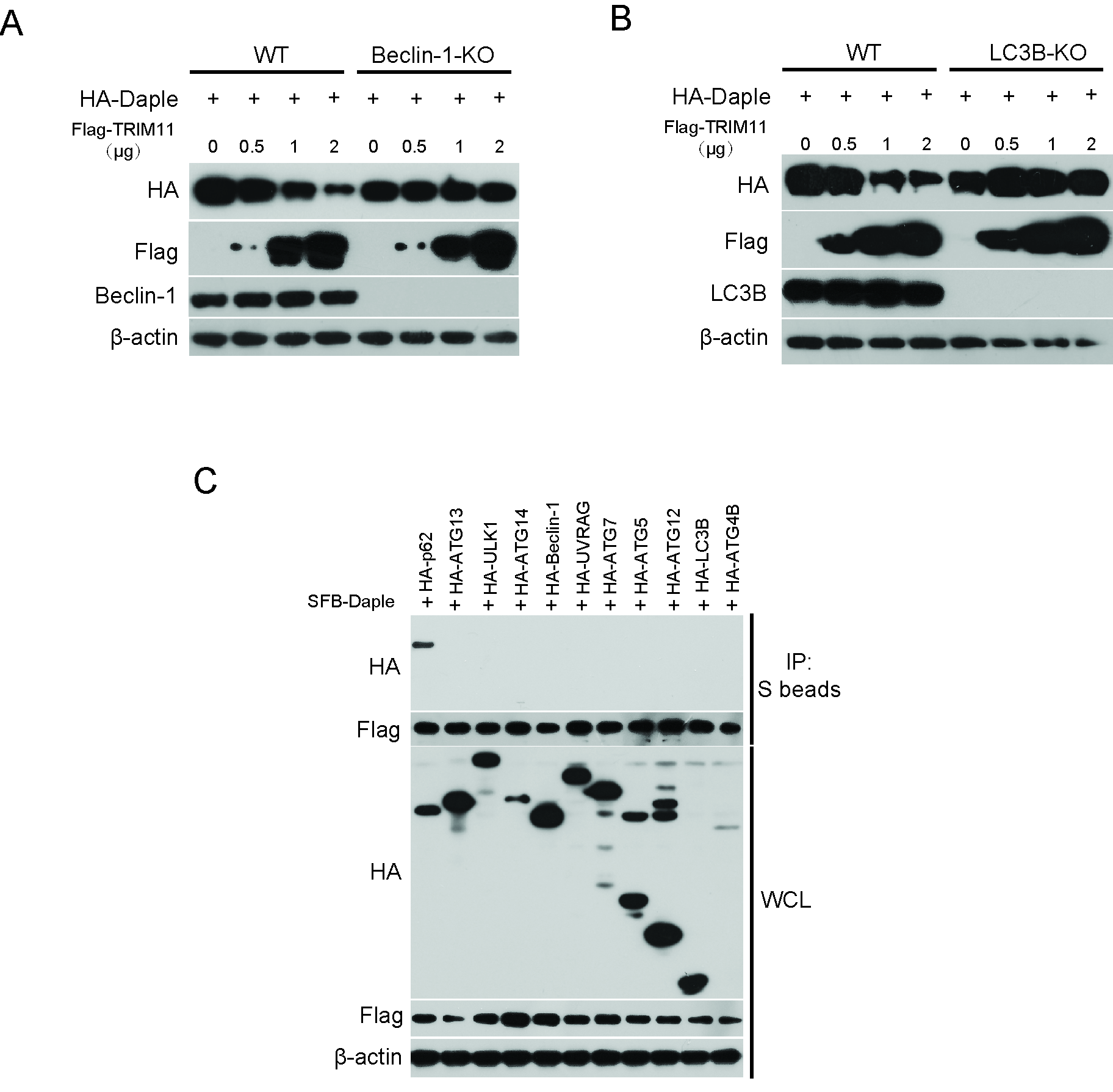


**Supplementary Figure 9.** (A) WT and BECN1 KO 293T cells were transfected with HA-Daple and increasing doses of plasmids for Flag-TRIM11 (wedge), and cell lysates were collected for immunoblotting. (B) WT and MAP1LC3B KO 293T cells were transfected with HA-Daple and increasing doses of plasmids for Flag-TRIM11, and cell lysates were collected for immunoblotting. (C) Co-IP and immunoblot analysis of 293T SFB-Daple cells cotransfected with empty vector for certain autophagic components, including HA-tagged p62, ATG13, ULK1, ATG14, Beclin-1, UVRAG, ATG7, ATG5, ATG12, LC3B or ATG4B.


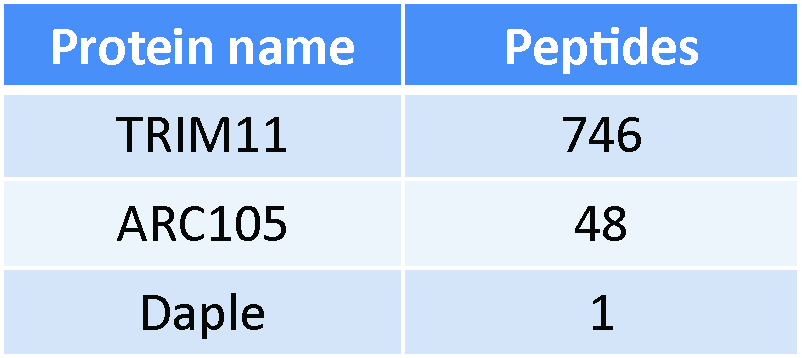


**Supplementary Table S4.** CNE2 cells stably expressing SFB-tagged (S-tag, Flag epitope tag, and streptavidin-binding peptide tag) TRIM11 were used for tandem affinity purification of protein complexes. Tables are summaries of proteins identified by mass spectrometry analysis.
