# Supplementary material for: TRIM11 facilitates chemoresistance in nasopharyngeal carcinoma by activating the β-catenin/ABCC9 axis via p62-selective autophagic degradation of Daple
Source: Oncogenesis. 2020 May 7;9(5):45. doi: 10.1038/s41389-020-0229-9 (PMC7206012; doi:10.1038/s41389-020-0229-9)
Supplement: Supplementary file 3 — Supplementary Tables 2 and 3 [file 41389_2020_229_MOESM3_ESM.docx]

**Supplementary Table S2: Clinical characteristics of 115 NPC patients**

| **Characteristics** | **No. (%)** |
| --- | --- |
| **Age, years** |  |
| Median | 47 |
| Range | 20-82 |
| **Sex** |  |
| Male | 88 (76.5) |
| Female | 27(23.5) |
| **Histological classification (WHO)** |  |
| Type II | 14(12.2.) |
| Type III | 101(87.8) |
| **Clinical staging** |  |
| I | 14(12.2) |
| II | 51(44.35) |
| III | 35(30.4) |
| IV | 15(13.05) |
| **Death** |  |
| Yes | 16（13.9） |
| No | 99（86.1） |

**Supplementary Table S3. Univariate and multivariate Cox regression analysis of TRIM11**

| **Variable** | **Univariate Cox regression** | | | **Multivariate Cox regression** | |
| --- | --- | --- | --- | --- | --- |
|  | **All cases** | **HR (95% CI)** | **P-*value*** | **HR (95% CI)** | **P-*value*** |
| **Age** |  |  |  |  |  |
| ≤47 | 60 | 1 | 0.165 |  |  |
| >47 | 55 | 0.473(0.164-1.361) |  |  |  |
| **Sex** |  |  |  |  |  |
| Male | 88 | 1 | 0.702 |  |  |
| Female | 27 | 0.783(0.223-2.748) |  |  |  |
| **Histological classification (WHO)** |  |  |  |  |  |
| Type II | 14 | 1 | 0.453 |  |  |
| Type III | 101 | 2.171(0.287-16.438) |  |  |  |
| **Clinical staging** |  |  |  |  |  |
| Early (I&II) | 65 | 1 | 0.002 | 1 | 0.019 |
| Late (III&IV) | 50 | 10.358(2.353-45.601) |  | 6.048(1.348-27.463) |  |
| **TRIM11 expression** |  |  |  |  |  |
| Low | 64 | 1 | 0.001 | 1 | 0.008 |
| High | 51 | 12.737(2.820-57.517) |  | 7.882(1.700-36.536) |  |
